# Iron deficiency linked to altered bile acid metabolism promotes *Helicobacter pylori*–induced inflammation–driven gastric carcinogenesis

**DOI:** 10.1172/JCI147822

**Published:** 2022-05-16

**Authors:** Jennifer M. Noto, M. Blanca Piazuelo, Shailja C. Shah, Judith Romero-Gallo, Jessica L. Hart, Chao Di, James D. Carmichael, Alberto G. Delgado, Alese E. Halvorson, Robert A. Greevy, Lydia E. Wroblewski, Ayushi Sharma, Annabelle B. Newton, Margaret M. Allaman, Keith T. Wilson, M. Kay Washington, M. Wade Calcutt, Kevin L. Schey, Bethany P. Cummings, Charles R. Flynn, Joseph P. Zackular, Richard M. Peek

**Affiliations:** 1 Division of Gastroenterology, Department of Medicine, Vanderbilt University Medical Center, Nashville, Tennessee, USA.; 2 Division of Protective Immunity, and; 3 Department of Biomedical and Health Informatics, Children’s Hospital of Philadelphia, Philadelphia, Pennsylvania, USA.; 4 Department of Biochemistry, Mass Spectrometry Research Center Laboratory, Vanderbilt University, Nashville, Tennessee, USA.; 5 Department of Biostatistics, Vanderbilt University Medical Center, Nashville, Tennessee, USA.; 6 Creighton University School of Medicine, Omaha, Nebraska, USA.; 7 Davidson College, Davidson, North Carolina, USA.; 8 Department of Veterans Affairs, Tennessee Valley Healthcare System, Nashville, Tennessee, USA.; 9 Department of Pathology, Microbiology, and Immunology, Vanderbilt University Medical Center, Nashville, Tennessee, USA.; 10 Department of Surgery, University of California, Davis, Davis, California, USA.; 11 Department of Surgery, Vanderbilt University Medical Center, Nashville, Tennessee, USA.; 12 Department of Pathology and Laboratory Medicine, University of Pennsylvania, Philadelphia, Pennsylvania, USA.

**Keywords:** Gastroenterology, Infectious disease, Bacterial infections, Gastric cancer, Mouse models

## Abstract

Gastric carcinogenesis is mediated by complex interactions among *Helicobacter pylori*, host, and environmental factors. Here, we demonstrate that *H*. *pylori* augmented gastric injury in INS-GAS mice under iron-deficient conditions. Mechanistically, these phenotypes were not driven by alterations in the gastric microbiota; however, discovery-based and targeted metabolomics revealed that bile acids were significantly altered in *H*. *pylori*–infected mice with iron deficiency, with significant upregulation of deoxycholic acid (DCA), a carcinogenic bile acid. The severity of gastric injury was further augmented when *H*. *pylori*–infected mice were treated with DCA, and, in vitro, DCA increased translocation of the *H*. *pylori* oncoprotein CagA into host cells. Conversely, bile acid sequestration attenuated *H*. *pylori*–induced injury under conditions of iron deficiency. To translate these findings to human populations, we evaluated the association between bile acid sequestrant use and gastric cancer risk in a large human cohort. Among 416,885 individuals, a significant dose-dependent reduction in risk was associated with cumulative bile acid sequestrant use. Further, expression of the bile acid receptor transmembrane G protein–coupled bile acid receptor 5 (TGR5) paralleled the severity of carcinogenic lesions in humans. These data demonstrate that increased *H*. *pylori*–induced injury within the context of iron deficiency is tightly linked to altered bile acid metabolism, which may promote gastric carcinogenesis.

## Graphical abstract

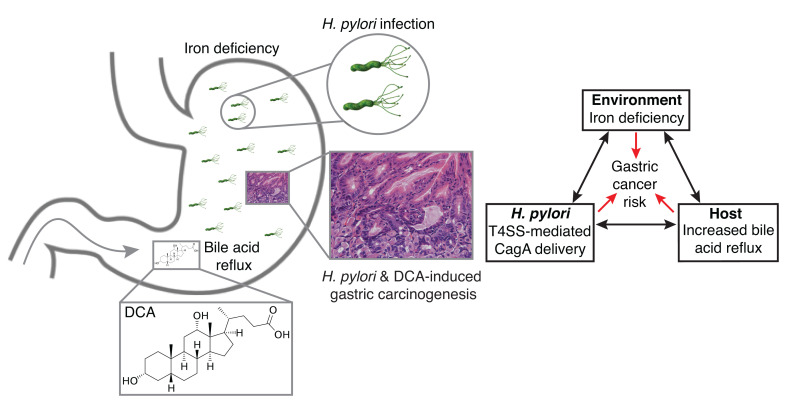

## Introduction

Gastric adenocarcinoma is the third leading cause of cancer-related mortality worldwide and accounts for more than 800,000 deaths annually (1). *Helicobacter pylori* is the most common bacterial infection worldwide and represents the strongest known risk factor for the development of gastric adenocarcinoma. Pathologic outcomes of infection are mediated by complex interactions among bacterial virulence determinants, host constituents, and environmental factors. One *H*. *pylori* strain–specific virulence determinant is the *cag* pathogenicity island. *H*. *pylori* strains that harbor the *cag* island induce more severe gastric injury and augment the risk for gastric cancer compared with *cag*-negative strains (2). The *cag* island encodes a bacterial type IV secretion system (T4SS), which translocates the effector protein CagA into host cells. Intracellular CagA can become phosphorylated (3–5) or remain unphosphorylated; in either form, CagA aberrantly activates numerous signaling pathways that are altered in gastric cancer, resulting in cellular responses that lower the threshold for carcinogenesis (6–8). The *cag* T4SS can also translocate peptidoglycan (9), heptose bisphosphate (10, 11), and microbial DNA into host cells (12). However, only a subset of persons infected by *cag*-positive *H*. *pylori* strains ever develop cancer (13, 14), underscoring the importance of defining additional factors that increase the risk for gastric cancer.

Environmental conditions also modify the risk for carcinogenesis. Iron deficiency is associated with an increased risk for neoplasms that arise within the stomach and the intestinal tract (15–17). Our laboratory previously demonstrated that iron deficiency augments and accelerates the development of gastric carcinogenesis within the context of *H*. *pylori* infection in the Mongolian gerbil model, which was mediated in part by increased function of the *cag* T4SS (18). The gerbil model of infection recapitulates many features of *H*. *pylori*–induced gastric inflammation and carcinogenesis in humans (19, 20); however, given the outbred nature of Mongolian gerbils, discernment of the precise role of host responses to *H*. *pylori* within the context of iron deficiency in this model has been limited. Therefore, the aim of this study was to define the specific effects of iron deficiency on host mediators of *H*. *pylori*–induced gastric inflammation and injury in 2 well-defined, independent, genetically inbred murine models of *H*. *pylori* infection: wild-type C57BL/6 mice and transgenic Friend virus B NIH FVB/N-Tg(Ins1-GAS) (FVB/N INS-GAS) mice that overexpress gastrin and are genetically predisposed to develop gastric dysplasia (21, 22). One limitation of using C57BL/6 mice to study gastric carcinogenesis is that the development of premalignant lesions requires prolonged periods of *H*. *pylori* colonization within the stomach (>12 months). In contrast, male transgenic hypergastrinemic INS-GAS mice develop preneoplastic lesions (21–24), which parallel features of human carcinogenesis, as early as 6 weeks following *H*. *pylori* challenge. In humans, *H*. *pylori*–induced pangastritis leads to hypochlorhydria, which predisposes to the development of gastric cancer (25). *H*. *pylori* infection can also cause hypergastrinemia in humans, mediated either by cytokine-induced stimulation of gastrin (26) or as a result of a positive feedback loop following parietal cell loss and hypochlorhydria. INS-GAS mice contain a human gastrin transgene, which results in overexpression of gastrin and leads to gastric hyperplasia and hypoacidity, accompanied by a steady decline in parietal cell mass. As such, uninfected INS-GAS mice spontaneously develop atrophic gastritis, dysplasia, and gastric adenocarcinomas at approximately 20 months of age. Following infection with *H*. *pylori*, gastric carcinogenesis is accelerated (21–24). Thus, the INS-GAS murine model parallels human gastric pathophysiology with respect to hypochlorhydria and hypergastrinemia, and, importantly, these mice develop advanced premalignant and malignant lesions that are not typically observed in wild-type mice. Host inflammatory and metabolic alterations that occur within gastric mucosa in response to *H*. *pylori* under conditions of iron deficiency were defined in these murine models and subsequently validated in humans.

## Results

### Iron deficiency augments H. pylori–induced gastric inflammation and injury in C57BL/6 and INS-GAS mice.

We used 2 independent inbred murine models of *H*. *pylori* infection — wild-type C57BL/6 mice and transgenic hypergastrinemic INS-GAS mice — to precisely define the effects of iron deficiency on host responses within the context of *H*. *pylori* infection. The mice were maintained on iron-replete or iron-depleted diets, challenged with or without the wild-type *cag*-positive *H*. *pylori* strain PMSS1, and then euthanized 8 weeks after challenge (Supplemental Figure 1A; supplemental material available online with this article; https://doi.org/10.1172/JCI147822DS1). To assess the effectiveness of iron depletion, we performed complete blood counts (CBCs) and assessed parameters of iron deficiency. Hemoglobin levels (Supplemental Figure 2 and Supplemental Figure 3, A and B) and hematocrit levels (Supplemental Figure 2 and Supplemental Figure 3, C and D) as well as mean corpuscular volume (Supplemental Figure 2 and Supplemental Figure 3, E and F) were significantly reduced among C57BL/6 and INS-GAS mice maintained on iron-depleted diets compared with those maintained on iron-replete diets, and this occurred independently of *H*. *pylori* infection (Supplemental Figures 2 and 3). Further, *H*. *pylori* infection did not induce iron deficiency or exacerbate the iron deficiency observed in mice maintained on an iron-depleted diet (Supplemental Figure 3).

Since gastric pH may affect the reduction of ferric iron (Fe^3+^) to ferrous iron (Fe^2+^), which could in turn affect iron absorption and further exacerbate iron deficiency, we next assessed the gastric pH in *H*. *pylori*–infected INS-GAS mice maintained on iron-replete or iron-depleted diets. The data demonstrated that chronically infected mice, regardless of diet, harbored a gastric pH of around 3–4 (data not shown), which suggests that iron would be available in a soluble and absorbable form and that pH levels would not further exacerbate the iron deficiency observed in this model.

**Figure 1:**
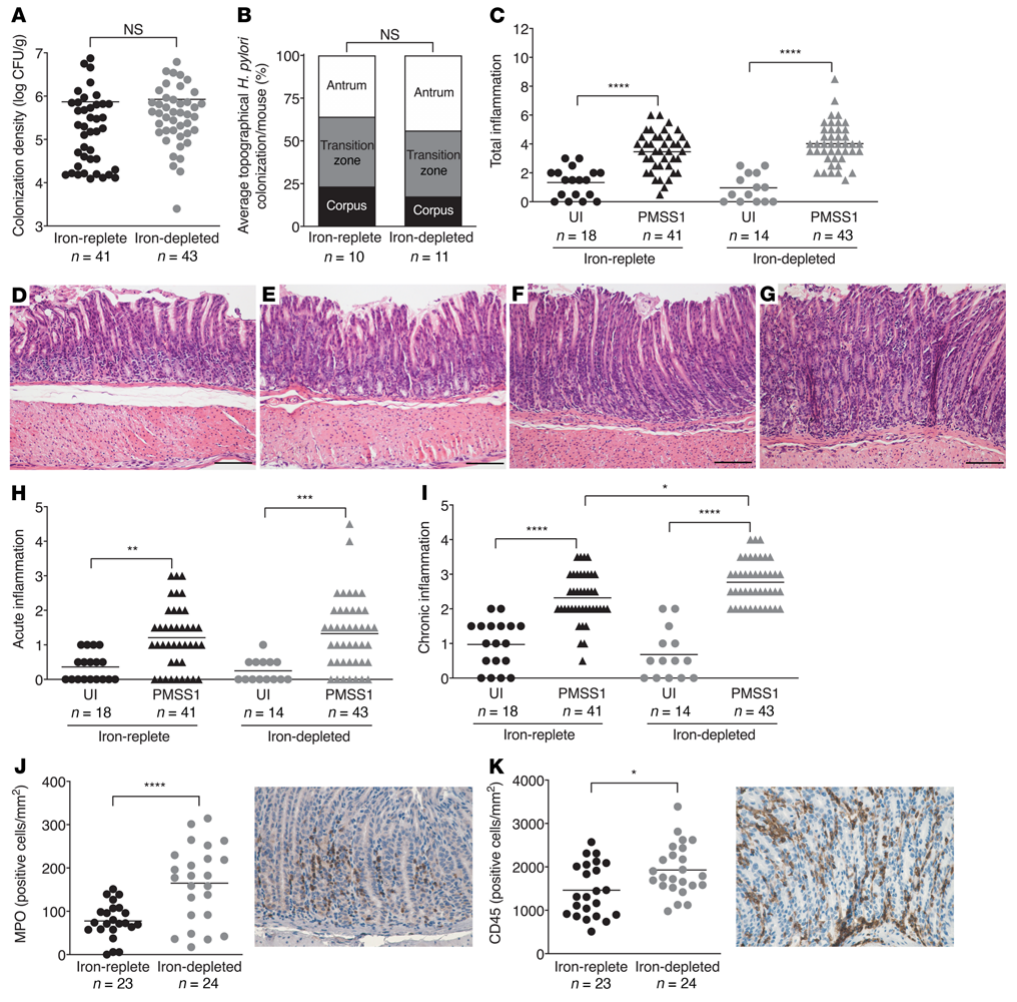
Iron deficiency augments *H*. *pylori*–induced chronic gastric inflammation in C57BL/6 mice. Wild-type male and female C57BL/6 mice were maintained on an iron-replete (*n* = 59) or iron-depleted (*n* = 57) diet and then challenged with Brucella broth (UI) or the *H*. *pylori* strain PMSS1. Mice were euthanized 8 weeks after challenge. (**A**) Gastric tissue was homogenized and plated for quantitative culturing. Colonization density is expressed as log CFU/g of tissue. (**B**) Gastric tissue was fixed, paraffin embedded, and stained with a modified Steiner stain. The percentage of *H*. *pylori* colonizing the antrum, transition zone, and corpus was assessed, and the average topographical *H*. *pylori* colonization density/mouse is shown. (**C**–**I**) Gastric tissue was fixed, paraffin embedded, and stained with H&E. The levels of total gastric inflammation (scored as 0–12) were assessed (**C**). (**D**–**G**) Representative histologic images from antrum of uninfected mice maintained on an iron-replete (**D**) or iron-depleted (**E**) diet and from *H*. *pylori*–infected mice maintained on an iron-replete (**F**) or iron-depleted (**G**) diet are shown (original magnification, ×200; scale bars: 100 μm). Tissue sections were scored separately for acute (score of 0–6) (**H**) and chronic gastric inflammation (score of 0–6) (**I**). Levels of MPO (**J**) and CD45 (**K**) were assessed by IHC to enumerate neutrophils and macrophages (MPO) and leukocytes (CD45), respectively. Representative images are shown (original magnification, ×400). Each point represents data from an individual animal from 3 independent experiments. Mean values are shown in the scatter dot plots. An unpaired parametric *t* test (**A**, **B**, **J**, and **K**) and 1-way ordinary ANOVA with Šidák’s multiple-comparison test (**C**, **H**, and **I**) were used to determine statistical significance. *P < 0.05, **P < 0.01, ***P < 0.001, and ****P < 0.0001.

Having established iron deficiency in our murine models, we next assessed *H*. *pylori* colonization and the severity of gastric inflammation and injury. Consistent with our previous findings in the Mongolian gerbil model, *H*. *pylori* colonization was not altered under conditions of iron deficiency in the C57BL/6 mice (Figure 1A). Further, we found that *H*. *pylori* colonized the gastric antrum, transition zone, and corpus at similar levels in mice maintained on either an iron-replete or iron-depleted diet (Figure 1B). We observed minimal infiltration of immune cells among uninfected mice maintained on either an iron-replete (Figure 1, C and D, and Supplemental Figure 4, A and E) or iron-depleted (Figure 1, C and E, and Supplemental Figure 4, B and E) diet, whereas inflammatory infiltrates were significantly increased among *H*. *pylori*–infected C57BL/6 mice maintained on either diet (Figure 1, C, F, and G, and Supplemental Figure 4, C–E). When we assessed the levels of acute and chronic inflammation independently, we found that *H*. *pylori* induced significantly higher levels of acute (Figure 1H) and chronic (Figure 1I) inflammation compared with uninfected mice, and chronic inflammation was further augmented under conditions of iron deficiency (Figure 1I). No differences in total, acute, or chronic gastric inflammation were observed among uninfected C57BL/6 mice maintained on either diet (Figure 1, C–I, and Supplemental Figure 4), indicating that iron deficiency augments gastric inflammation only within the context of *H*. *pylori* infection. To more rigorously assess the extent and severity of gastric inflammation among C57BL/6 mice, we quantitatively assessed levels of myeloperoxidase (MPO) (Figure 1J) and CD45 (Figure 1K) by IHC to enumerate neutrophils and macrophages (MPO) as well as leukocytes (CD45), respectively. These data parallel the inflammation scores and demonstrate that iron deficiency significantly augmented the infiltration of immune cells and *H*. *pylori*–induced inflammation.

**Figure 2:**
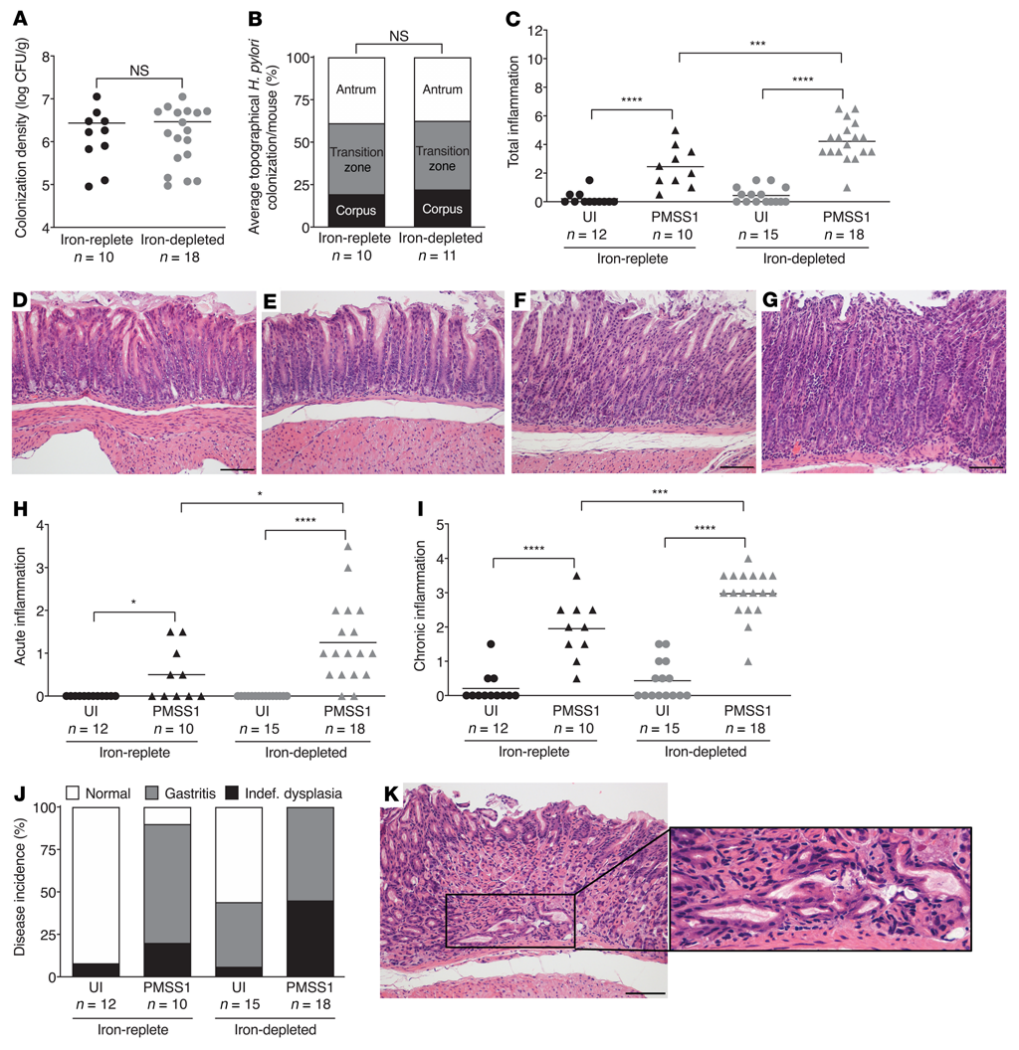
Iron deficiency augments *H*. *pylori*–induced gastric inflammation and injury in INS-GAS mice. Male INS-GAS mice were maintained on an iron-replete (*n* = 22) or iron-depleted (*n* = 33) diet and then challenged with Brucella broth (UI) or the *H*. *pylori* strain PMSS1. Mice were euthanized 8 weeks after challenge. (**A**) Gastric tissue was homogenized and plated for quantitative culturing. Colonization density is expressed as log CFU/g of tissue. (**B**) Gastric tissue was fixed, paraffin embedded, and stained with a modified Steiner stain. The percentage of *H*. *pylori* colonizing the antrum, transition zone, and corpus was assessed, and the average topographical *H*. *pylori* colonization density/mouse is shown. (**C**–**K**) Gastric tissue was fixed, paraffin embedded, and stained with H&E. (**C**) Levels of total gastric inflammation (score of 0–12). (**D**–**G**) Representative histologic images from the antrum of uninfected mice maintained on an iron-replete (**D**) or iron-depleted (**E**) diet and of *H*. *pylori*–infected mice maintained on an iron-replete (**F**) or iron-depleted (**G**) diet (original magnification, ×200; scale bars: 100 μm). Gastric tissue was scored separately for acute (**H**) and chronic (**I**) inflammation and disease incidence (**J**). Dysplasia was graded as indefinite dysplasia (borderline nuclear and architectural epithelial changes that do not completely fit the patterns of dysplasia), low-grade or high-grade dysplasia. (**K**) Representative histologic images of indefinite dysplasia. Original magnification, ×200 and ×400 (enlarged inset). Scale bar: 100 μm. Each point represents data from an individual animal from 3 independent experiments. Mean values are shown in the scatter dot plots. An unpaired parametric *t* test (**A** and **B**), 1-way ordinary ANOVA with Šidák’s multiple-comparison test (**C**, **H**, and **I**), and Fisher’s exact test (**J**) were used to determine statistical significance. *P < 0.05, ***P < 0.001, and ****P < 0.0001.

To validate and extend these findings to a more robust model of gastric carcinogenesis, INS-GAS mice were maintained on an iron-replete or iron-depleted diet and then challenged with the *H*. *pylori* strain PMSS1 (Supplemental Figure 1A). As with the C57BL/6 mice, we found that *H*. *pylori* colonization was not altered under conditions of iron deficiency (Figure 2A) and that mice maintained on iron-replete or iron-depleted diets had similar levels of *H*. *pylori* colonization in the gastric antrum, transition zone, and corpus (Figure 2B). *H*. *pylori* also induced significantly higher levels of total inflammation in INS-GAS mice maintained on iron-replete and iron-depleted diets compared with uninfected control mice, and this was significantly augmented under conditions of iron deficiency (Figure 2, C–G, and Supplemental Figure 5).

When the levels of acute and chronic inflammation were assessed independently, both acute (Figure 2H) and chronic (Figure 2I) inflammation were further exacerbated under iron-depleted conditions. Consistent with heightened levels of gastric inflammation among infected INS-GAS mice maintained on an iron-depleted diet, the incidence of gastric dysplasia was also heightened, whereby 44% of infected mice maintained on an iron-depleted diet developed gastric dysplasia compared with 20% of infected mice maintained on an iron-replete diet (Figure 2, J and K).

### H. pylori–induced inflammation and injury under conditions of iron deficiency are reversible.

**Figure 3:**
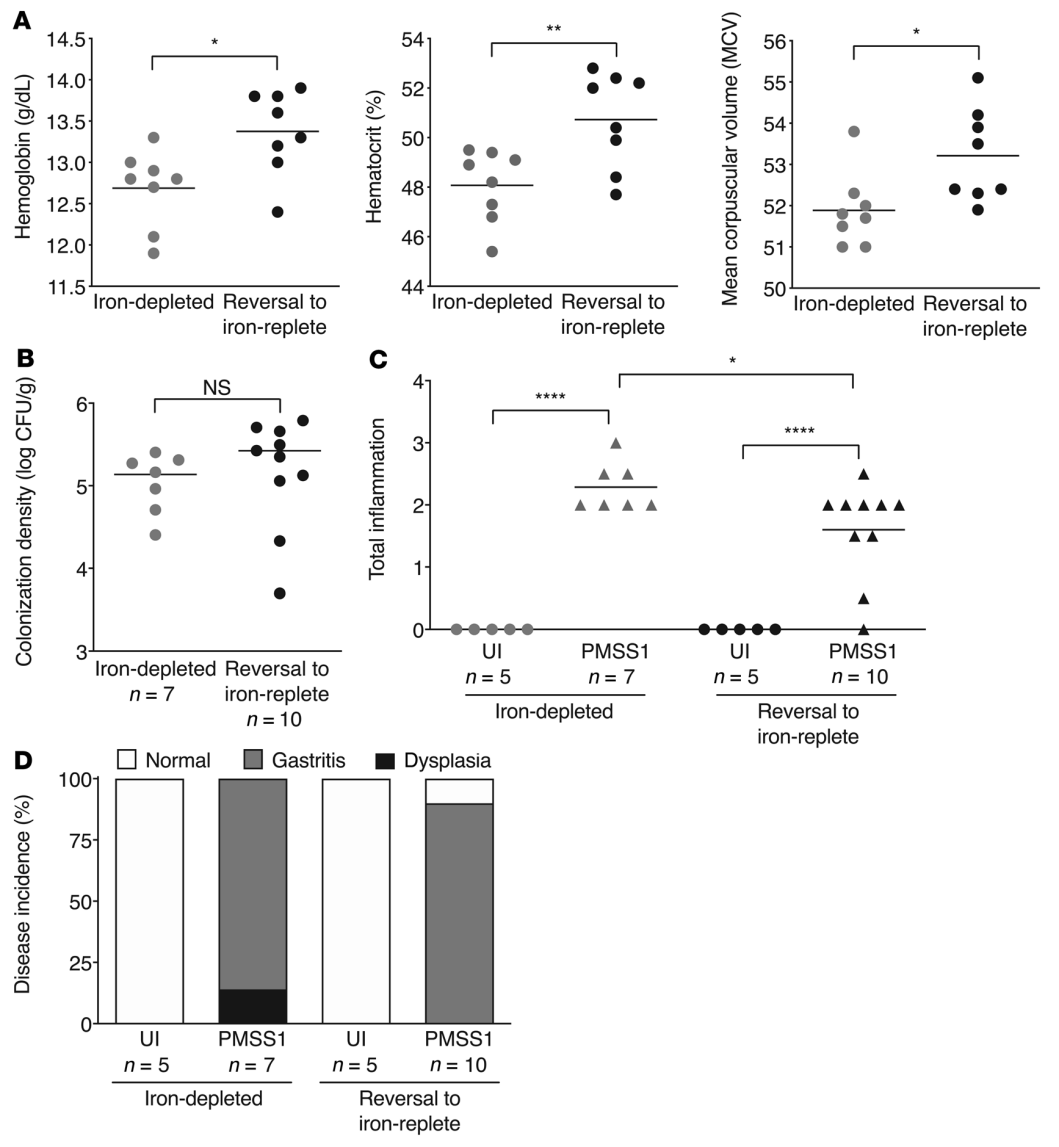
*H*. *pylori*–induced inflammation and injury under conditions of iron deficiency is reversible. Two groups of male transgenic hypergastrinemic INS-GAS mice were maintained on a iron-depleted diet and then challenged with Brucella broth (UI) or the *H*. *pylori* strain PMSS1. Two weeks after challenge, 1 group was continued on an iron-depleted diet (*n* = 12) and the other group was switched to an iron-replete diet (*n* = 15). The mice were euthanized 8 weeks after challenge. (**A**) Blood was harvested from a subset of mice for CBC analysis. Hemoglobin, hematocrit, and mean corpuscular volume were assessed as parameters of iron deficiency. (**B**) Gastric tissue was homogenized and plated for quantitative culturing. Colonization density is expressed as log CFU/g of tissue. (**C**) Gastric tissue was fixed, paraffin embedded, and stained with H&E. Total gastric inflammation levels (score of 0–12) were assessed. (**D**) Gastric tissue was also scored for disease incidence. Disease incidence included normal histopathology, gastritis, and gastric dysplasia. Dysplasia was graded as indefinite dysplasia, low-grade dysplasia, or high-grade dysplasia. Each point represents data from an individual animal from 2 independent experiments. Mean values are shown in the scatter dot plots. An unpaired parametric *t* test (**A** and **B**), 1-way ordinary ANOVA with Šidák’s multiple-comparison test (**C**), and Fisher’s exact test (**D**) were used to determine statistical significance. *P < 0.05, **P < 0.01, and ****P < 0.0001.

To assess whether *H*. *pylori*–induced inflammatory phenotypes observed under conditions of iron deficiency in INS-GAS mice could be reversed, 2 groups of mice were maintained on iron-depleted diets for 2 weeks prior to challenge with or without the *H*. *pylori* strain PMSS1. One group was continued on an iron-depleted diet, while the other group was switched to an iron-replete diet 2 weeks after challenge. The mice were then euthanized 8 weeks after challenge (Supplemental Figure 1B). Consistent with our previous findings, the parameters of iron deficiency were significantly reduced among mice maintained on an iron-depleted diet, but were restored following the switch to an iron-replete diet (Figure 3A). *H*. *pylori* colonization density was similar among mice maintained on an iron-depleted diet compared with mice switched to an iron-replete diet (Figure 3B). Importantly, the severity of *H*. *pylori*–induced inflammation and injury was significantly reduced when the mice were switched from an iron-depleted diet to an iron-replete diet (Figure 3, C and D), suggesting that these phenotypes are reversible following restoration of iron status.

### H. pylori induces biased proinflammatory immune responses within the context of iron deficiency.

**Figure 4:**
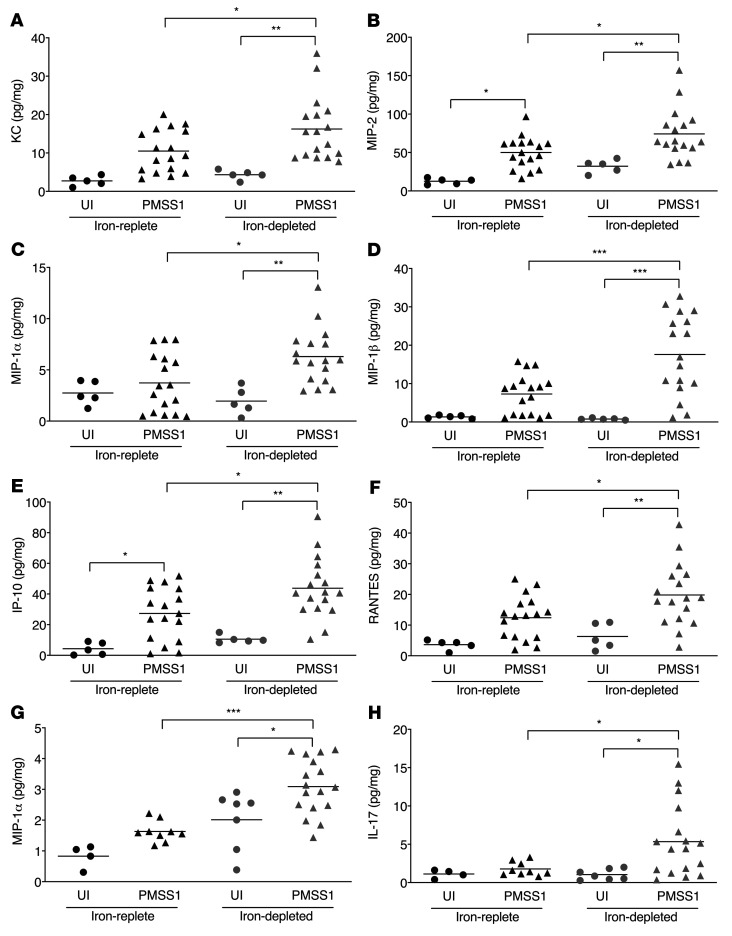
*H*. *pylori* induces proinflammatory responses in C57BL/6 and INS-GAS mice within the context of iron deficiency. Male and female C57BL/6 and male INS-GAS mice were maintained on an iron-replete or iron-depleted diet and then challenged with Brucella broth (UI) or the *H*. *pylori* strain PMSS1. Mice were euthanized 8 weeks after challenge. Gastric tissue was analyzed using a cytokine/chemokine multiplex bead array. Data were acquired and analyzed using the Millipore software platform and expressed as picograms of chemokine per milligram of gastric tissue. Levels of KC (**A**), MIP-2 (**B**), MIP-1α (**C**), MIP-1β (**D**), IP-10 (**E**), and RANTES (**F**) were significantly increased by *H*. *pylori* infection in C57BL/6 mice under conditions of iron deficiency compared with infected mice maintained on an iron-replete diet. Levels of MIP-1α (**G**) and IL-17 (**H**) were significantly increased in *H*. *pylori*–infected INS-GAS mice under conditions of iron deficiency compared with infected mice maintained on an iron-replete diet. Each point represents data from an individual animal from 3 independent experiments. C57BL/6 mice: iron-replete UI (*n* = 5) and PMSS1 (*n* = 17); iron-depleted UI (*n* = 5) and PMSS1 (*n* = 17). INS-GAS mice: iron-replete UI (*n* = 4) and PMSS1 (*n* = 9); iron-depleted UI (*n* = 7) and PMSS1 (*n* = 17). Mean values are shown in the scatter dot plots. A 1-way ordinary ANOVA with Šidák’s multiple-comparison test was used to determine statistical significance. Only statistically significant comparisons are denoted. *P < 0.05, **P < 0.01, and ***P < 0.001.

We next sought to define specific mechanisms that regulate iron-deficient phenotypes in a subset of C57BL/6 and INS-GAS mice using a 25-plex chemokine and cytokine panel. As expected, we observed a significant increase in the levels of several proinflammatory chemokines and cytokines following *H*. *pylori* infection, some of which were further augmented under conditions of iron deficiency. Among C57BL/6 mice, KC (Figure 4A) and MIP-2 (Figure 4B), chemokines important for neutrophil recruitment, MIP-1α (Figure 4C) and MIP-1β (Figure 4D), chemokines important for directing macrophage responses, and IP-10 (Figure 4E) and RANTES (Figure 4F), chemokines important in driving Th1 T cell responses, were significantly increased among infected mice under conditions of iron deficiency compared with infected mice on an iron-replete diet. Several other cytokines were increased in infected C57BL/6 mice but were not significantly altered by iron deficiency. These included IL-1α, IL-1β, IL-2, and IL-17 (Supplemental Figure 6). Among INS-GAS mice, MIP-1α (Figure 4G) and IL-17 (Figure 4H) were significantly increased in infected mice under conditions of iron deficiency compared with infected mice on an iron-replete diet. Several other cytokines, including IP-10, IL-1α, KC, INF-γ, MIP-2, IL-1β, IL-6, and IL-15, were also increased among INS-GAS mice infected with *H*. *pylori*, independent of iron status (Supplemental Figure 7).

### In vivo adaptation of H. pylori to conditions of iron deficiency does not alter cag T4SS function or vacA expression.

We previously demonstrated that in vivo adaptation of *H*. *pylori* to conditions of iron deficiency in Mongolian gerbils led to increased function of the *cag* T4SS, as determined by CagA expression and translocation (18). Thus, to assess the effects of iron deficiency on *cag* T4SS function in mice, we isolated in vivo–adapted *H*. *pylori* strains from C57BL/6 and INS-GAS mice maintained on either an iron-replete or iron-depleted diet. *H*. *pylori* strains were cocultured with gastric epithelial cells, and CagA expression levels and CagA translocation, as measured by tyrosine phosphorylation, were assessed by Western blot analysis (Supplemental Figure 8). In contrast to Mongolian gerbils, we found that in vivo–adapted strains isolated from either C57BL/6 or INS-GAS mice did not differ in their ability to express CagA (Supplemental Figure 8, A–D) or translocate CagA into host cells (Supplemental Figure 8, A, B, and E–H) following adaptation to either dietary condition.

To assess whether in vivo adaptation to conditions of iron deficiency altered the expression levels of another well-characterized *H*. *pylori* virulence factor, vacuolating cytotoxin (*vacA*), we isolated in vivo–adapted *H*. *pylori* strains from C57BL/6 and INS-GAS mice maintained on either an iron-replete or iron-depleted diet. RNA was extracted from minimally passaged, log-phase *H*. *pylori*, and *vacA* gene expression was assessed by quantitative reverse transcription PCR (qRT-PCR). In vivo–adapted *H*. *pylori* strains isolated from either C57BL/6 (Supplemental Figure 9A) or INS-GAS (Supplemental Figure 9B) mice did not exhibit significant differences in *vacA* gene expression following adaptation to either dietary condition. Collectively, these results indicate that host-driven mechanisms may mediate iron-dependent phenotypes in mice.

### The gastric microbiota is not a significant driver of the proinflammatory phenotypes among INS-GAS mice under iron deficiency.

To first assess the potential effect of the gastrin transgene on the microbiota, we harvested gastric tissue from uninfected wild-type FVB/N mice and transgenic hypergastrinemic INS-GAS mice (Supplemental Figure 1C) for 16S rRNA gene sequencing and microbial community analysis. These data showed that there were no differences in α-diversity (Supplemental Figure 10A) between FVB/N and INS-GAS mice, but subtle differences in β-diversity (Supplemental Figure 10B), which were driven by the differential abundance of a sole genus, *Turicibacter* (Supplemental Figure 10C). These data suggest that the gastrin transgene does not significantly alter the diversity of the gastric microbiota.

To determine the potential effects of iron deficiency on the microbiota, we harvested gastric tissue from uninfected INS-GAS mice maintained on an iron-replete or iron-depleted diet (Supplemental Figure 1C) for 16S rRNA gene sequencing and microbial community analysis. These data revealed no differences in α-diversity (Supplemental Figure 10D) or β-diversity (Supplemental Figure 10E) between INS-GAS mice maintained on an iron-replete versus those on an iron-depleted diet, indicating that iron deficiency does not significantly or dramatically alter the diversity or the community structure of the gastric microbiota. Collectively, these data suggest that gastric microbiota are probably not a significant driver of the proinflammatory phenotypes in INS-GAS mice observed under conditions of iron deficiency. However, we cannot definitively exclude a potential effect of individual species or microbe-derived metabolites in the gastric microbiota or the effect of the intestinal microbiota, which would require more in-depth analysis and possibly long-term monocolonization studies.

### H. pylori alters metabolic profiles in C57BL/6 and INS-GAS mice under conditions of iron deficiency.

**Figure 5:**
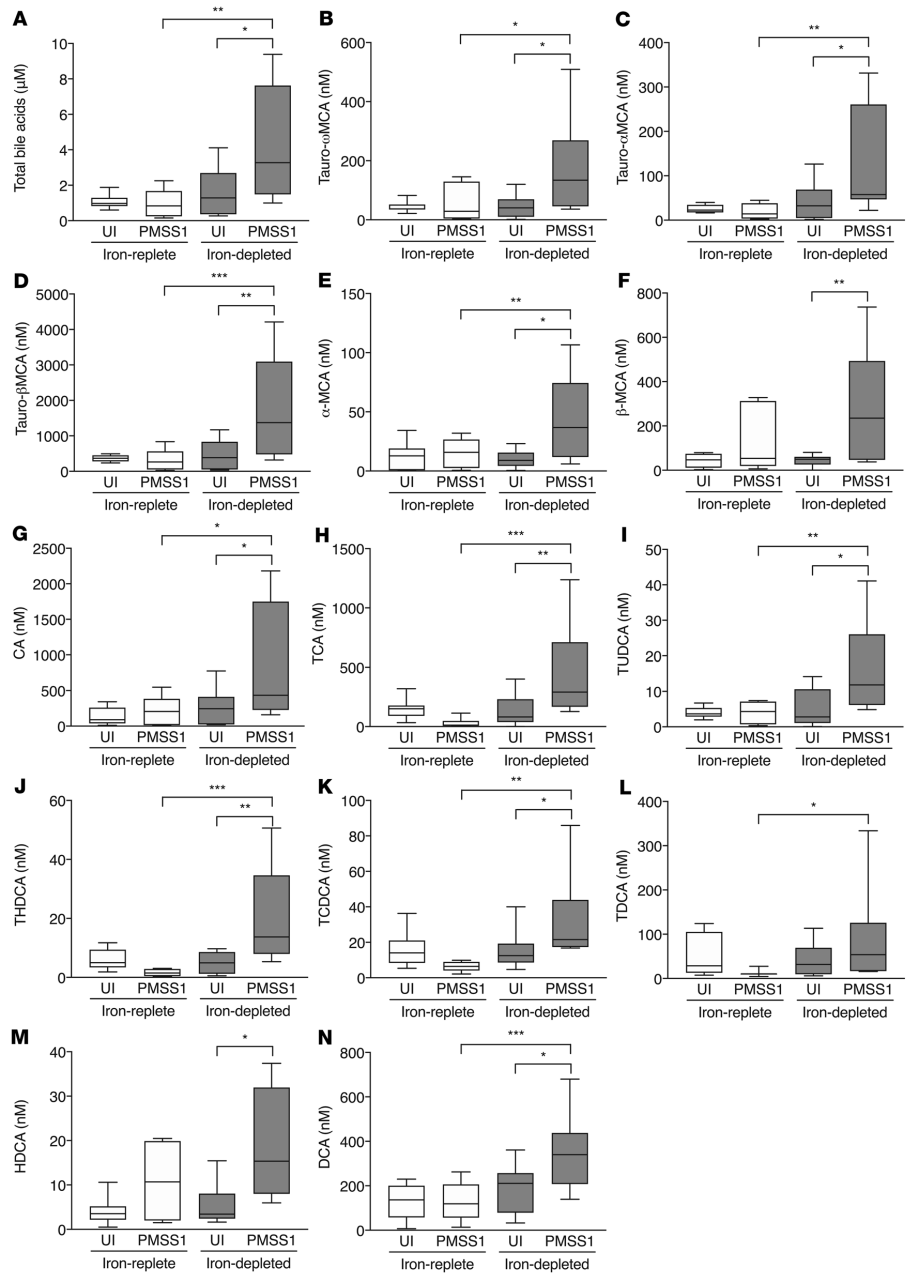
*H*. *pylori* significantly alters bile acid levels under conditions of iron deficiency in INS-GAS mice. Male INS-GAS mice were maintained on an iron-replete or iron-depleted diet and then challenged with Brucella broth or the wild-type *H*. *pylori* strain PMSS1. Mice were euthanized 8 weeks after challenge. Gastric tissue was processed for targeted bile acid analyses. Total bile acids (**A**), muricholic acids (**B**–**F**), cholic acids (**G** and **H**), and DCAs (**I**–**N**) were significantly increased by *H*. *pylori* under conditions of iron deficiency compared with infected mice under iron-replete conditions. *n* = 10 mice analyzed per group from 2 independent experiments. Median values are shown in box-and-whisker plots, with whiskers designating minimum and maximum values. A 1-way ordinary ANOVA with Šidák’s multiple-comparison test was used to determine statistical significance. Only statistically significant comparisons are denoted. *P < 0.05, **P < 0.01, and ***P < 0.001.

To increase the mechanistic depth of our studies, we next performed untargeted metabolomics studies on gastric tissue isolated from uninfected or *H*. *pylori*–infected C57BL/6 and INS-GAS mice maintained on either an iron-replete or iron-depleted diet. Comprehensive lists of the identified gastric mucosal metabolites in C57BL/6 mice and INS-GAS mice are provided in Supplemental Tables 1 and 2, respectively. From these individual metabolites, 4 major metabolic pathways were predicted to be altered following infection with *H*. *pylori* in both models: arginine and polyamine metabolism, l-lysine and purine degradation, fatty acid metabolism, and bile acid metabolism. On the basis of previous data demonstrating that bile acids are linked to gastrointestinal malignancies (27–29), we subsequently conducted targeted bile acid analyses in both C57BL/6 (Supplemental Figure 11) and INS-GAS (Figure 5) mice.

Among C57BL/6 mice, we found that total bile acid levels were significantly increased following *H*. *pylori* infection under conditions of iron deficiency (Supplemental Figure 11A). However, there were no significant differences in variants of muricholic acid (MCA) or cholic acid (CA) following *H*. *pylori* infection under iron-replete or iron-depleted conditions (Supplemental Figure 11 B–H). Tauroursodeoxycholic acid (TUDCA) was significantly increased following *H*. *pylori* infection under conditions of iron deficiency (Supplemental Figure 11I), while taurohyodeoxycholic acid (THDCA) was significantly increased following *H*. *pylori* infection under iron-replete conditions (Supplemental Figure 11J). We detected no significant differences in deoxycholic acid (DCA) levels (Supplemental Figure 11N).

Among INS-GAS mice with a predisposition to carcinogenesis, bile acid levels were minimally changed under iron-replete conditions, when uninfected mice were compared with *H*. *pylori*–infected mice (Figure 5). However, total bile acids were significantly increased under conditions of iron deficiency in conjunction with *H*. *pylori* infection (Figure 5A), similar to what we observed in C57BL/6 mice. However, in contrast to C57BL/6 mice, variants of MCA, CA, and DCA were significantly increased by *H*. *pylori* under conditions of iron deficiency compared with infected mice under iron-replete conditions (Figure 5, B–N). Collectively, these data indicate that iron deficiency, in conjunction with *H*. *pylori* infection, is linked to altered bile acid metabolism in INS-GAS mice. These data raise the hypothesis that bile acids may mediate the increased gastric inflammation and injury observed in this model.

### DCA treatment augments H. pylori–induced gastric inflammation and injury under iron-depleted conditions in INS-GAS mice.

**Figure 6:**
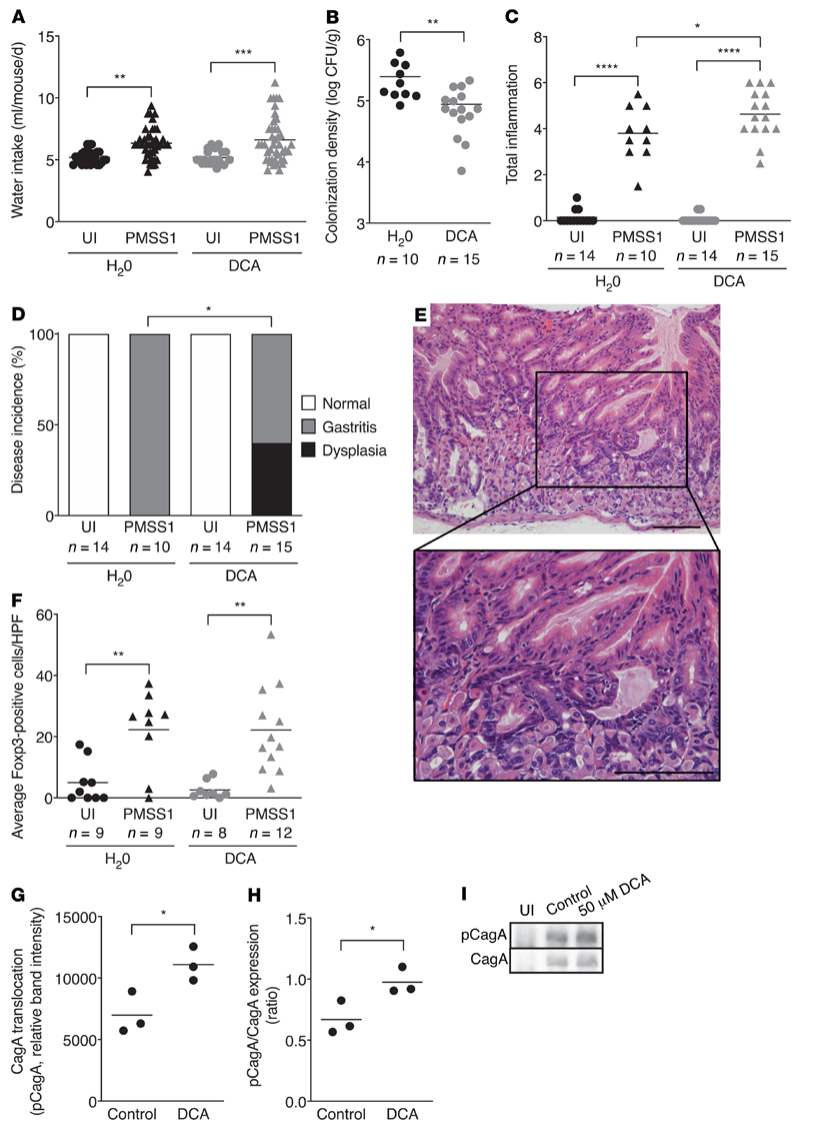
DCA treatment significantly augments *H*. *pylori*–induced gastric inflammation and injury. Male INS-GAS mice were maintained on an iron-replete standard diet and then challenged with Brucella broth (*n* = 28) or the *H*. *pylori* strain PMSS1 (*n* = 25). Two weeks after infection, mice received water alone or water supplemented with 100 μM DCA throughout the course of the experiment. Mice were euthanized 6 weeks after challenge. (**A**) Average water consumption was measured. (**B**) Gastric tissue was harvested for quantitative culturing. Colonization density is expressed as log CFU/gram of tissue. Gastric tissue was assessed for indices of gastric inflammation (**C**) and disease incidence (**D**). Disease incidence includes normal histopathology, gastritis, and gastric dysplasia. Dysplasia was graded as indefinite dysplasia, low-grade dysplasia, or high-grade dysplasia. (**E**) Representative histologic image of low-grade dysplasia. Original magnification, ×200 and ×400 (enlarged inset). Scale bars: 100 μm. (**F**) The average number of Foxp3-positive cells was assessed by IHC from 5 high-powered fields (×400). Each point represents data from an individual animal from 3 independent experiments. (**G**–**I**) Gastric epithelial cells were cocultured with the *H*. *pylori* strain PMSS1 and then treated with either vehicle control or 50 μM DCA for 6 hours, and protein lysates were harvested for Western blot analysis. Levels of phosphorylated CagA (**G**) and the ratio of phosphorylated CagA (pCagA) to total CagA (**H**). Representative Western blots (**I**). Mean values are shown in the scatter dot plots. An unpaired parametric *t* test (**B**, **G**, and **H**), 1-way ordinary ANOVA with Šidák’s multiple-comparison test (**A**, **C**, and **F**), and Fisher’s exact test (**D**) were used to determine statistical significance. Only statistically significant comparisons are denoted. *P < 0.05, **P < 0.01, ***P < 0.001, and ****P < 0.0001.

Bile acids regulate inflammation (30) and promote gastrointestinal malignancies, including premalignant and malignant lesions of the stomach (31–33). DCA and other secondary bile acids have also been demonstrated to increase the production of ROS and induce DNA damage, which increase the risk of carcinogenesis (34–36). To directly assess the effects of the secondary bile acid DCA on the development of *H*. *pylori*–induced gastric inflammation and injury, INS-GAS mice were maintained on an iron-replete standard diet, challenged with or without *H*. *pylori*, and were then provided water (H_2_0) alone or water supplemented with 100 μM DCA (Supplemental Figure 1D). Of interest, *H*. *pylori*–infected mice consumed significantly more water than uninfected mice, but there were no differences in the consumption of water alone versus water supplemented with 100 μM DCA (Figure 6A). Neither *H*. *pylori* infection nor the consumption of water supplemented with 100 μM DCA led to differences in parameters of iron deficiency, including serum ferritin, hemoglobin, and hematocrit levels, or mean corpuscular volume (Supplemental Figure 12). *H*. *pylori* colonization was significantly lower in mice receiving water supplemented with 100 μM DCA compared with those that received water alone (Figure 6B). *H*. *pylori* significantly increased gastric inflammation among mice receiving water alone or water supplemented with DCA compared with that seen in uninfected mice, and this was further augmented in mice receiving DCA (Figure 6C). Consistent with increased levels of inflammation, DCA also significantly increased the incidence of low-grade gastric dysplasia compared with infected mice receiving water alone (40% versus 0%, Figure 6, D and E), suggesting that secondary bile acids, such as DCA, may play a direct role in mediating *H*. *pylori*–induced inflammation and injury. Bacterial bile acid metabolism plays a key role in modulating innate immunity by controlling the balance of inflammatory Th cells and antiinflammatory Tregs in intestinal tissue. Secondary bile acids have been shown to induce Tregs by increasing Foxp3 expression (37–39). Thus, we assessed the expression of Foxp3 by IHC in gastric tissue sections from uninfected and *H*. *pylori*–infected mice treated with or without DCA. These results demonstrate that *H*. *pylori* induced the expression of Foxp3 in gastric tissue, but DCA alone did not alter the abundance of Foxp3-positive cells in the stomach, suggesting that the presence of secondary bile acids may exacerbate gastric inflammation rather than promote an antiinflammatory Treg response (Figure 6F).

Since DCA has been shown to induce secretion system function and virulence in other bacterial pathogens, such as *Shigella* (40), we next assessed the direct role of DCA in *H*. *pylori* Cag T4SS function. Gastric epithelial cells were cocultured with *H*. *pylori* and either vehicle control or DCA, and the levels of CagA expression and translocation were assessed by Western blot analysis. The data showed that *H*. *pylori* exposure to DCA induced a significant increase in the translocation of CagA into host cells (Figure 6, G–I), indicating that augmentation of Cag T4SS function is another mechanism regulating increased inflammation and injury by DCA under conditions of iron deficiency.

To assess and extend the role of *H*. *pylori* and DCA in an acute model of infection, we converted 3D human gastric organoids to 2D monolayers, cocultured them with or without *H*. *pylori*, and subsequently treated them with or without DCA (Supplemental Figure 13). Compared with the uninfected controls (Supplemental Figure 13A), *H*. *pylori* infection (Supplemental Figure 13B), DCA treatment (Supplemental Figure 13C), and the combination of *H*. *pylori* infection and DCA treatment (Supplemental Figure 13C) induced increased levels of EGFR activation. *H*. *pylori* infection, DCA treatment, and the combination of *H*. *pylori* and DCA also induced significantly higher levels of IL-8 production and epithelial cell proliferation compared with uninfected controls (Supplemental Figure 13, E and F). Collectively, these data indicate that DCA induces the activation of proinflammatory and proliferative signaling pathways that may facilitate gastric carcinogenesis.

### Bile acid sequestration significantly attenuates H. pylori–induced inflammation and injury under conditions of iron deficiency.

**Figure 7:**
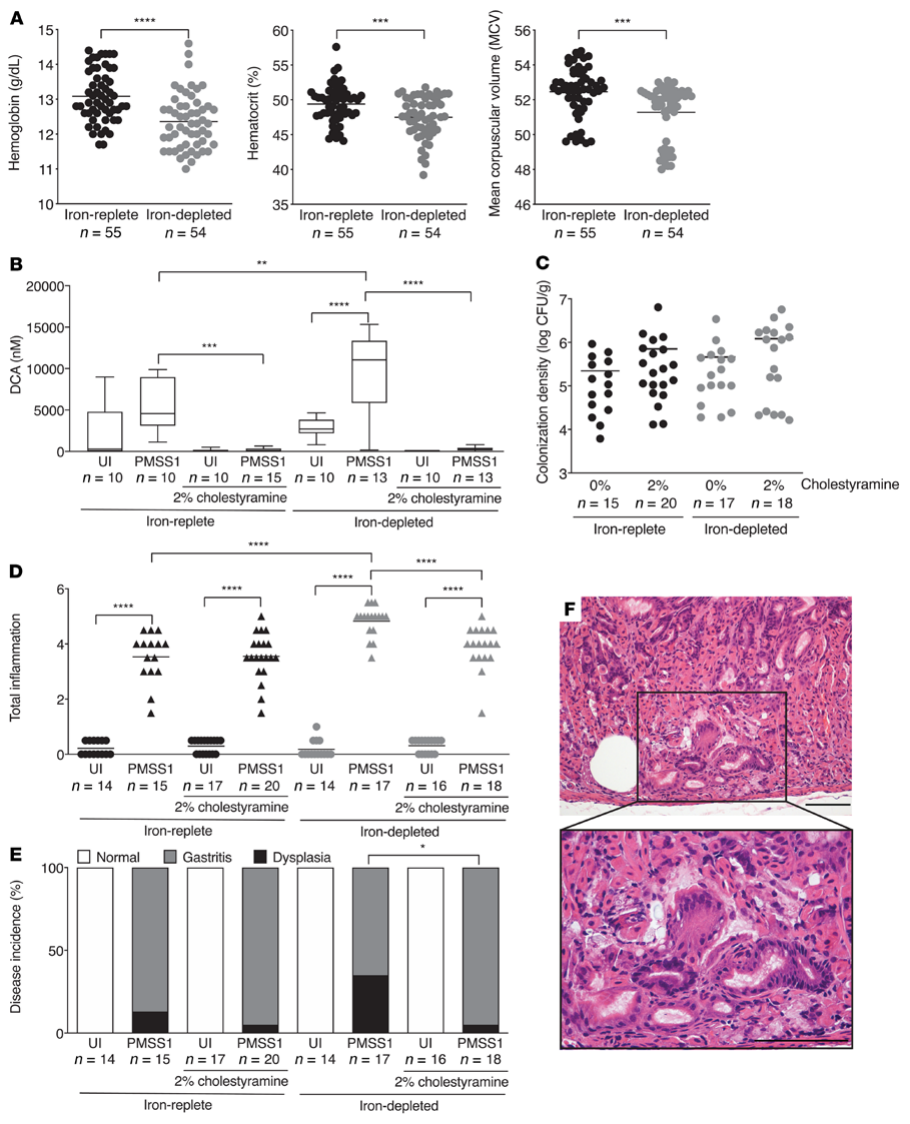
Cholestyramine treatment significantly reduces *H*. *pylori*–induced inflammation and injury under conditions of iron deficiency in INS-GAS mice. Male INS-GAS mice were maintained on an iron-replete (*n* = 66) or iron-depleted (*n* = 65) diet supplemented with or without 2% cholestyramine (w/w) and then challenged with Brucella broth or the *H*. *pylori* strain PMSS1. Mice were euthanized 8 weeks after challenge. Whole blood was collected for CBC analysis from a subset of *H*. *pylori*–infected mice maintained on an iron-replete or iron-depleted diet with or without cholestyramine supplementation. (**A**) Hemoglobin, hematocrit, and mean corpuscular volume were assessed as parameters of iron deficiency. (**B**) Gastric tissue was processed for targeted bile acid analyses to assess DCA levels. (**C**) Gastric tissue was harvested for quantitative culturing. Colonization density is expressed as log CFU/g of tissue. (**D**–**F**) Gastric tissue was assessed for indices of inflammation (**D**) and disease incidence (**E**). Disease incidence includes normal histopathology, gastritis, and gastric dysplasia. Dysplasia was graded as indefinite dysplasia (borderline nuclear and architectural epithelial changes that do not completely fit the patterns of dysplasia), low-grade dysplasia, or high-grade dysplasia. (**F**) Representative histologic image of indefinite dysplasia is shown. Original magnification, ×200 and ×400 (enlarged inset). Scale bars: 100 μm. Each point represents data from an individual animal from 3 independent experiments. Mean values are shown in the scatter dot plots. Median values are shown in box-and-whisker plots, with whiskers designating minimum and maximum values. An unpaired parametric *t* test (**A**), 1-way ordinary ANOVA with Šidák’s multiple-comparison test (**B**–**D**), and Fisher’s exact test (**E**) were used to determine statistical significance. Only statistically significant comparisons are denoted. *P < 0.05, **P < 0.01, ***P < 0.005, and ****P < 0.0001.

To conversely assess the effects of a bile acid sequestrant on the development of *H*. *pylori*–induced gastric inflammation and injury under conditions of iron depletion, male INS-GAS mice were maintained on an iron-replete or iron-depleted diet supplemented with or without 2% cholestyramine, challenged with or without *H*. *pylori*, and then euthanized 8 weeks after challenge (Supplemental Figure 1E). As expected, hemoglobin and hematocrit levels and mean corpuscular volume (Figure 7A) were significantly reduced in the *H*. *pylori*–infected mice maintained on an iron-depleted diet with or without cholestyramine, compared with the *H*. *pylori*–infected mice maintained on an iron-replete diet with or without cholestyramine, and cholestyramine had no effect on the parameters of iron deficiency (Supplemental Figure 14). To assess the effectiveness of cholestyramine treatment, we performed targeted bile acid analysis, which showed that cholestyramine treatment significantly reduced the secondary bile acid DCA in gastric tissue (Figure 7B). The mice exhibited similar levels of *H*. *pylori* colonization when maintained on an iron-replete or iron-depleted diet, regardless of the presence of cholestyramine (Figure 7C).

Having confirmed the effectiveness of an iron-depleted diet, we next assessed the levels of gastric inflammation and injury. As expected, we found that infection with *H*. *pylori* significantly increased gastric inflammation in mice under both iron-replete and iron-depleted conditions compared with uninfected controls, and this was significantly augmented under conditions of iron deficiency (Figure 7D). Importantly, treatment with cholestyramine significantly reduced the levels of *H*. *pylori*–induced gastric inflammation under conditions of iron deficiency (Figure 7D). Consistent with our previous findings (Figure 2F), iron deficiency increased the incidence of dysplasia in *H*. *pylori*–infected mice. However, consistent with reduced *H*. *pylori*–induced inflammation, cholestyramine treatment also significantly reduced the incidence of gastric dysplasia under conditions of iron deficiency (35% versus 5%, Figure 7, E and F). Collectively, these results suggest that bile acids augment *H*. *pylori*–induced inflammation and injury under conditions of iron deficiency in INS-GAS mice and that bile acid-reducing therapies may represent an effective means of controlling detrimental host responses.

### Use of bile acid sequestrants is associated with a reduced risk of gastric cancer in humans.

To extend our in vivo studies to human populations and to test the hypothesis that decreased gastric mucosal exposure to bile acids is associated with a reduced risk of gastric cancer, we conducted a retrospective cohort analysis of the association between bile acid sequestrant medications and the risk of incident gastric cancer. Using a national database containing electronic health records with individual-level data (*n* = ~15 million), including prescription medication fills and longitudinal medical follow-up, we constructed a cohort of individuals who had undergone testing for *H*. *pylori* (*n* = 725,134). We selected this as the base cohort in order to limit selection and ascertainment bias, as well as to reduce residual confounding at inception. In this database, electronic health records are accurate and complete starting from the year 2000 onward. After exclusion of patients (see Supplemental Methods), the final analytic data set included 416,885 patients. The date of study entry was defined as the date of *H*. *pylori* testing. Among those individuals who met the full inclusion criteria for the analytic cohort, all completed prescription fills of bile acid sequestrant medications in the 5 years prior to study entry through the last date of follow-up were captured for each patient (primary exposure). Cumulative exposure to bile acid sequestrant medications was a continuous variable representing the proportion of the total number of days exposed to bile acid sequestrants. The total number of days of exposure was determined by calculating the quantity dispensed for individual bile acid sequestrant prescriptions. Specific bile acid sequestrants included cholestyramine, colestipol, and colesevelam. The mean follow-up time for the cohort was 7.9 years (SD: 4 years). Therefore, including the 5 years prior to study entry, cumulative exposure to bile acid sequestrants was captured over approximately 13 years for this analysis.

The demographics and clinical characteristics of the individuals comprising the analytic cohort are provided in Table 1. There were 19,634 (4.7%) individuals who had some exposure to bile acid sequestrants at any time prior to study entry or during the follow-up period. As is characteristic of veteran demographics, the majority of the cohort were men (89%) and non-Hispanic White individuals (63%). Given the large size of the cohort (*n* = 416,885), the small differences in covariates between exposed and nonexposed groups, while statistically significant (by Wilcoxon test or Pearson’s χ^2^ test), may not be clinically significant. For example, a 1% difference in the frequency of male sex and a 2% difference in *H*. *pylori* prevalence are both statistically significant according to the Pearson’s χ^2^ test *P* value of less than 0.05, but these are likely not clinically significant differences. Compared with individuals who had no exposure to bile acid sequestrants, those with exposure tended to be slightly older (mean age [SD]: 57 [14] versus 54 [16] years), slightly less often male (88% versus 89%), more often non-Hispanic White (70% versus 63%), more often smokers (44% versus 37%), and slightly less often *H*. *pylori* positive (32% versus 34%). Among the 416,885 individuals, a total of 1956 cases of gastric cancer occurred during follow-up. After adjusting for relevant confounders, bile acid sequestrant use on 1% of the total days was associated with a significant 8% reduced risk of incident gastric cancer (adjusted HR, 0.92; 95% CI, 0.86–0.98; *P* < 0.01; Table 2). This reduction in risk was greater with a larger proportion of total days of exposure; bile acid sequestrant exposure on 5% of the total days was associated with a significant 30% risk reduction (HR, 0.70; 95% CI, 0.53–0.92; *P* = 0.015; Table 2). This observation appeared to be maintained with an increased proportion of 20% days of exposure (HR, 0.71; 95% CI, 0.48–1.04; *P* = 0.073; Table 2), although the smaller sample size in this stratum limited the power. When the reference category was bile acid sequestrant exposure for 1% of total days (instead of no exposure), exposure on 5% of the days was associated with a significant 24% reduced risk of incident gastric cancer (HR, 0.76; 95% CI, 0.62–0.94; *P* < 0.05; data not shown). When analyzed separately, cholestyramine exposure on 5% of the total days compared with no exposure had a slightly greater protective effect (HR, 0.51; 95% CI, 0.30–0.86; *P* < 0.05; data not shown); however, the reduced sample size precludes strong conclusions. The suggestive protective effect of bile acid sequestrants on the risk of incident gastric cancer was also maintained, irrespective of the anatomic location of the gastric cancer.

### Expression of the bile acid receptor TGR5 parallels the severity of gastric disease in humans.

**Figure 8:**
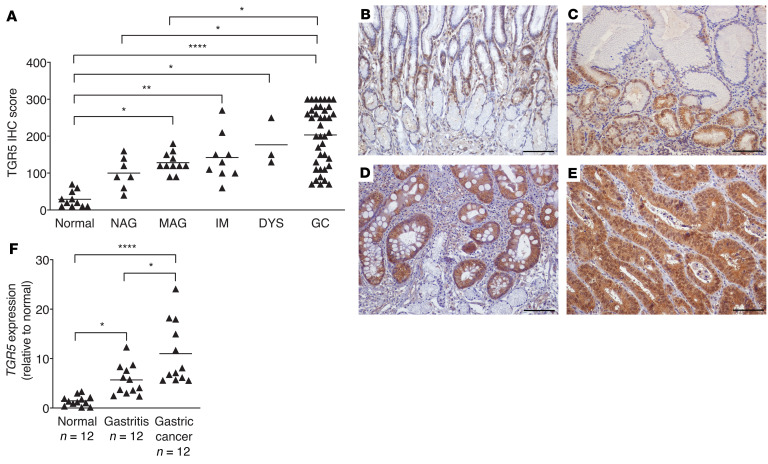
TGR5 expression parallels the severity of gastric disease. (**A**) TGR5 protein expression was evaluated by IHC in human gastric tissues from a gastric cancer TMA. A single pathologist assessed the percentage of TGR5-positive cells and the intensity of TGR5 staining. The IHC score reflects the percentage of cells positive for TGR5, multiplied by the intensity of staining, as previously described (61). TGR5 staining was assessed in gastric tissue sections from patients with no pathology (normal, *n* = 11), nonatrophic gastritis (NAG, *n* = 7), multifocal atrophic gastritis (MAG, *n* = 11) without intestinal metaplasia, intestinal metaplasia (IM, *n* = 9), dysplasia (DYS, *n* = 3), or gastric cancer (GC, *n* = 40). (**B**–**E**) Representative images of TGR5 protein expression in normal gastric tissue sections (**B**) and gastric tissue sections with multifocal atrophic gastritis (**C**), intestinal metaplasia (**D**), and gastric cancer (**E**). Original magnification, ×200. Scale bars: 100 μm. (**F**) RNA was extracted from normal gastric tissue (*n* = 12), gastric tissue with gastritis alone (*n* = 12), and gastric tissue with gastric adenocarcinoma (*n* = 12). *TGR5* mRNA expression levels were standardized to *GAPDH* mRNA expression levels and are shown as fold relative to normal [2^–(ΔΔCt)]. Mean values are shown in the scatter dot plots. A 1-way ordinary ANOVA with Šidák’s multiple-comparison test was used to determine statistical significance. Only statistically significant comparisons are denoted. *P < 0.05, **P < 0.01, and ****P < 0.0001.

Transmembrane G protein–coupled bile acid receptor 5 (TGR5) is an important bile acid receptor that regulates bile acid metabolism and mucosal immune homeostasis. TGR5 is expressed in parenchymal cells as well as in hematopoietic cell lineages, and, among bile acid receptors, TGR5 has the highest affinity for secondary bile acids (30, 41). Importantly, TGR5 has been shown to be highly expressed in gastric cancer, with expression levels being associated with increased proliferation, migration, and epithelial-mesenchymal transition (42) and decreased patient survival (43). To further investigate the role of bile acid–mediated pathogenesis in humans and identify potentially druggable targets, we used a human gastric tissue microarray (TMA) to assess TGR5 levels in patients with varying disease pathologies across the cascade of gastric carcinogenesis. We found that TGR5 expression paralleled the severity of gastric disease, with the highest levels present in patients with noncardia gastric cancer (Figure 8A). There were no differences in TGR5 expression levels between diffuse versus intestinal-type gastric cancer (data not shown). TGR5 staining patterns increased in a step-wise manner from normal gastric tissue (Figure 8B) to multifocal atrophic gastritis without intestinal metaplasia (Figure 8C), to intestinal metaplasia (Figure 8D), to gastric cancer (Figure 8E). To validate these findings, we next measured *TGR5* expression via qRT-PCR in normal human gastric tissue, gastric tissue with gastritis alone, and gastric tissue with adenocarcinoma (Table 3). These data corroborate our IHC findings and demonstrate that *TGR5* gene expression levels parallel the severity of gastric lesions (Figure 8F).

To explore the role of *Tgr5* in *H*. *pylori*–induced gastric inflammation in mice in greater depth, we challenged wild-type C57BL/6 and *Tgr5^–/–^* homozygous littermates with Brucella broth, as an uninfected control, or with the *H*. *pylori* strain PMSS1 and then euthanized the mice 8 weeks after challenge (Supplemental Figure 1F). *H*. *pylori* colonization density (Supplemental Figure 15A) and *H*. *pylori*–induced inflammation levels (Supplemental Figure 15B) were not significantly different between *H*. *pylori*–infected wild-type mice and *Tgr5^–/–^* mice, indicating that TGR5 may either represent a biomarker for disease severity in humans or that TGR5 may have different roles in promoting disease in humans and mice.

## Discussion

*H*. *pylori* induces detrimental host responses that lower the threshold for the development of gastric cancer. Using refined and tractable genetic models, we have demonstrated that *H*. *pylori* increased the severity of gastric inflammation and the development of premalignant lesions in the setting of iron deficiency, and this was, in part, mediated through altered bile acid production and a concomitant increase in levels of the secondary bile acid DCA, which has been associated with a variety of gastrointestinal cancers (27–29, 34). Treatment with DCA directly contributed to and exacerbated *H*. *pylori*–induced inflammation and the development of premalignant lesions, while a targeted reduction of bile acids through treatment with cholestyramine significantly reduced *H*. *pylori*–induced inflammation (*P* < 0.0001) and injury (*P* < 0.05) in the context of iron deficiency. Importantly, in a large human cohort with confirmed *H*. *pylori* testing, the use of bile acid sequestrant medications was associated with a significantly (*P* < 0.05) reduced risk of gastric cancer based on cumulative exposure over time. Although this correlation between bile acid sequestrant use and risk of gastric cancer is important, there is a crucial need for this to be studied in the future in a more controlled fashion in a population with a higher incidence of gastric cancer.

Bile acids are present predominantly in the bile of mammals and are synthesized from cholesterol in the liver. Primary bile acids can be conjugated with glycine or taurine to form primary conjugated bile acids, which are then secreted via the bile duct into the duodenum, where they emulsify and solubilize lipid-soluble nutrients to facilitate digestion and absorption of cholesterol and lipids. In the colon, primary bile acids serve as a substrate for bacterial biotransformation into secondary bile acids, such as DCA and lithocholic acid, which can exert pathogenic effects under certain conditions. The majority of intestinal bile acids are reabsorbed from the terminal ileum and transported back to the liver through the process of enterohepatic recirculation. Previous reports demonstrated that high levels of bile acids are present in the gastric juice of healthy individuals (44) and patients with ulcer disease (45). Further, the combination of bile reflux and acid reflux increases the risk of gastritis, which has been linked to gastric cancer in a recent large human cohort study (46). Another recent study characterized differences in gastric bile acid composition between patients with gastritis and healthy individuals and demonstrated that although bile acids are present in the stomachs of healthy individuals and patients with non–bile acid reflux gastritis, conjugated bile acids were prominent components in the gastric juices of patients with bile acid reflux gastritis (47). Collectively, these studies demonstrate that bile acid reflux occurs in patients with or without gastritis, but that increased levels of conjugated bile acids are associated with the risk for gastric carcinogenesis.

In addition to their role as dietary surfactants, bile acids serve as key signaling molecules in the modulation of epithelial cell proliferation, gene expression, and metabolism. However, when such homeostatic pathways are disrupted, bile acids can promote inflammation, metabolic disorders, and cancer. Specifically, secondary, hydrophobic bile acids function as tumor promoters for a variety of gastrointestinal cancers (27–29, 34) through a combination of mechanisms, including induction of direct oxidative stress with concomitant DNA damage, proliferation, apoptosis, epigenetic gene regulation, altered expression of bile acid receptors, and dysbiosis of the gut microbiota. The secondary bile acid, DCA, can induce activation of the transcription factor NF-κB, leading to increased inflammatory cytokine production, enhanced cell survival, and hyperproliferation in esophageal (48, 49), gastric (50), and colon (51) cancer cells. DCA can also induce COX-2 expression through transactivation of the EGFR (52), which leads to inflammation and hyperproliferation in pancreatic (53) and squamous esophageal (54) cells. In addition to transactivation of EGFR, DCA can induce epithelial hyperproliferation through activation of ERK and PKC signaling pathways (55) and can also activate β-catenin signaling, ERK1 and ERK2 signaling via activator protein 1 (AP1), and c-Myc–targeted pathways (56), stimulating cancer cell proliferation and invasiveness. In addition to direct interactions with transmembrane and nuclear signaling receptors on epithelial cells, bile acids also activate innate and adaptive immune cells to regulate mucosal immune responses (30, 57, 58). Our new data now reveal a previously unappreciated role that secondary bile acids may play in *H*. *pylori* virulence, namely, augmented function of the Cag type IV secretion system. Thus, dysregulation of bile acid homeostasis leads to the activation of numerous deleterious host and microbial signaling pathways directly involved in heightened proinflammatory responses and the activation of signaling cascades that predispose to and promote carcinogenesis.

Bile acid–mediated signaling occurs through 2 primary bile acid receptors: farsenoid X receptor (FXR) and TGR5, both of which are important for regulating bile acid metabolism and mucosal immune homeostasis (30, 59). The FXR is expressed on parenchymal cells, primarily hepatocytes and intestinal epithelial cells, whereas TGR5 is expressed on these cells as well as on hematopoietic lineage cells, most notably monocytes and macrophages. Importantly, TGR5 has the highest affinity for secondary bile acids (30, 41) and has been shown to be overexpressed in gastric adenocarcinomas, and increased expression of TGR5 is strongly associated with decreased patient survival (43). Further, overexpression of TGR5 is associated with increased proliferation, migration, and epithelial-mesenchymal transition in gastric cancer cells (MKN45) and human gastric adenocarcinomas (42). We have now demonstrated that TGR5 expression levels also significantly increased with advancing disease severity along the gastric carcinogenic cascade. The discovery of the role of bile acid receptors in a number of diseases, including cholangiocarcinoma, hepatocellular carcinoma, and colon cancer, has led to the development of novel receptor agonists for potential treatments and therapies (41). Our data therefore indicate that, in addition to heightened levels of bile acids, TGR5 expression is increased in gastric carcinogenesis in humans and thus may be an important prognostic factor for gastric cancer. However, studies are needed to provide more direct evidence linking TGR5 receptor signaling to gastric carcinogenesis, such as those performed in animal models of *Tgr5* deficiency that develop more advanced gastric disease in response to *H*. *pylori* infection.

In summary, our studies demonstrate that iron deficiency augmented gastric carcinogenesis in the context of *H*. *pylori* infection and this was, in part, mediated through altered bile acid metabolism. These findings raise the intriguing possibility that targeting bile acid metabolic pathways and/or bile acid receptor signaling to reduce the levels of specific bile acids and the accumulation of secondary bile acids as well as inhibiting the activation of detrimental signaling pathways may be a beneficial treatment strategy for gastric cancer prevention. Quantification of the levels of iron, detrimental secondary bile acids, and bile acid receptors in *H*. *pylori*–infected individuals may also be useful in identifying individuals at high risk for progression to gastric cancer.

## Methods

### Murine models of iron deficiency and H. pylori infection.

Male and female wild-type C57BL/6 (Envigo) and male transgenic hypergastrinemic INS-GAS^+/+^ mice on a FVB/N background (21–24) were bred and housed in the Vanderbilt University Medical Center animal care facilities in a room with a 12-hour light/12-hour dark cycle, maintained at 21°C–22°C. Rodent diets were modified from TestDiet AIN-93M (Purina Feed) to contain 0 ppm iron (iron-depleted, TestDiet 5TWD) or 250 ppm iron (iron-replete, TestDiet 5STQ). Mice were maintained on modified diets for 2 weeks prior to challenge with *H*. *pylori* and throughout the duration of the experiments. The wild-type *cag*-positive *H*. *pylori* strain PMSS1 was minimally passaged on trypticase soy agar plates with 5% sheep’s blood (TSA/SB, BD Biosciences) and in Brucella broth (BD Biosciences) supplemented with 10% FBS (Atlanta Biologicals) for 16 hours at 37°C with 5% CO_2_. Mice were orogastrically challenged with Brucella broth, as an uninfected (UI) negative control, or with wild-type *cag*-positive *H*. *pylori* strain PMSS1 (Supplemental Figure 1A). For the diet reversal experiment, 2 groups of male INS-GAS mice were maintained on an iron-depleted diet 2 weeks prior to challenge with *H*. *pylori*. The mice were then challenged with Brucella broth or the wild-type *cag*-positive *H*. *pylori* strain PMSS1. Two weeks after challenge, 1 group of mice was continued on an iron-depleted diet, while the other group of mice was switched to an iron-replete diet. The mice were euthanized 8 weeks after challenge (Supplemental Figure 1B). To assess the potential effect of the gastrin transgene on the gastric microbiota, gastric tissue was harvested from uninfected male FVB/N (Envigo) and INS-GAS mice that had been co-housed for 8 weeks (Supplemental Figure 1C). To assess the potential effect of iron deficiency on the gastric microbiota, gastric tissue was harvested from uninfected male INS-GAS mice that were co-housed and then maintained on an iron-replete or iron-depleted diet for 8 weeks (Supplemental Figure 1C). For DCA treatment, male INS-GAS mice were maintained on an iron-replete standard rodent diet (LabDiet, PicoLab Laboratory Rodent Diet, 5L0D*) and then orogastrically challenged with Brucella broth or the *H*. *pylori* strain PMSS1. Two weeks after the challenge, the mice were provided water alone or water supplemented with 100 μM DCA (MilliporeSigma) and were euthanized 6 weeks after challenge (Supplemental Figure 1D). For cholestyramine-mediated bile acid suppression, male INS-GAS mice were maintained on an iron-replete or iron-depleted diet with or without 2% cholestyramine (MilliporeSigma, w/w) for 2 weeks prior to challenge with *H*. *pylori* and throughout the course of the experiment. Mice were then orogastrically challenged with Brucella broth or the *H*. *pylori* strain PMSS1 and euthanized 8 weeks after challenge (Supplemental Figure 1E).

### Histopathology.

Linear strips of gastric tissue, extending from the squamocolumnar junction through the proximal duodenum, were fixed in 10% neutral-buffered formalin (Azer Scientific) and then paraffin embedded and stained with H&E. A pathologist, blinded to the treatment groups, assessed the indices of inflammation and the incidence of gastric injury, including gastric dysplasia. The severity of acute and chronic inflammation was graded from 0–3 in both the gastric antrum and corpus, as previously described (18), for a cumulative score of 0–12. Dysplasia was graded as indefinite dysplasia (borderline nuclear and architectural epithelial changes that do not completely fit the patterns of dysplasia), low-grade dysplasia, or high-grade dysplasia.

### IHC analysis.

To assess Foxp3 protein expression in mice, IHC analysis was performed on formalin-fixed, paraffin-embedded (FFPE) gastric tissue from INS-GAS mice using a rat monoclonal anti-Foxp3 antibody (Invitrogen, Thermo Fisher Scientific, catalog 14-577-82). The number of Foxp3-positive cells was counted in 5 high-powered fields (HPFs, ×400) with the highest counts for each mouse, and the average number of Foxp3-positive cells per HPF is shown. To assess MPO and CD45 protein expression in mice, IHC analysis was performed on FFPE gastric tissue from C57BL/6 mice using rabbit polyclonal anti-MPO (prediluted, Biocare Medical, PP023AA) and anti-CD45 (Abcam ab10558) antibodies, respectively. MPO- and CD45-positive cells were enumerated throughout the entire length of the mucosa and submucosa in each mouse and quantified as the number of positive cells per square millimeter of mucosa and submucosa. QuPath software was used to assist with the analysis of digital images of entire slides (60). To assess TGR5 protein expression in humans, IHC analysis was performed on a gastric cancer TMA using a rabbit polyclonal anti-GPCR TGR5 antibody (Abcam ab72608). The gastric cancer TMA was generated at Vanderbilt University Medical Center with tissues from the Cooperative Human Tissue Network. A single pathologist scored TGR5 IHC staining in the gastric sections. The percentage of positive cells was assessed, and the intensity of epithelial staining was graded on a scale of 0–3 (absent = 0, weak = 1, moderate = 2, strong = 3). IHC data are represented as TGR5 IHC scores, which were determined by multiplying the staining intensity by the percentage of positively stained cells, as previously described (61).

### Statistics.

Mean values are shown with the SEM, scatter dot plots are shown with mean values, and box-and-whisker plots are shown with median values, with whiskers designating minimum and maximum values. An unpaired parametric *t* test, 1-way ordinary ANOVA with Šidák’s multiple-comparison test, χ^2^ test, and Fisher’s exact test were used for statistical comparisons. No samples were excluded from the analyses. All statistical analyses were performed using GraphPad Prism and a *P* value of less than 0.05 was considered statistically significant.

For the human retrospective cohort analysis, descriptive characteristics of individuals with bile acid sequestrant exposure versus nonexposure were generated and then compared using Pearson’s χ^2^ test for categorical variables and Wilcoxon test for continuous variables. The date of study entry (*t_0_*, time zero) was defined as the date of *H*. *pylori* testing. Individuals were followed until the earliest of the following events: incident gastric cancer diagnosis (primary outcome), death, loss to follow-up, or end of study period (May 31, 2020). Multivariable Cox regression analysis was performed to estimate the association between cumulative bile acid medication exposure and risk of incident gastric cancer. Secondary analyses of individual bile acid sequestrant medications as the primary exposure were performed, and secondary analyses with the primary outcome as incident gastric cancer according to anatomic location were also conducted. We generated three categories of cumulative exposure based on the distribution of individuals’ proportion of total days of exposure to bile acid sequestrant medications (continuous variable) in order to assess whether the association with risk of gastric cancer changed according to magnitude of cumulative exposure. These categories were graded as 1% of total days exposed to 5% of total days exposed to greater than 20% of total days exposed. Analyses were adjusted for relevant confounders, including age, sex, race/ethnicity, smoking, and *H*. *pylori* status. All statistical analyses were performed using Stata 15 and R. Statistical significance was set at a 2-tailed *P* value of less than 0.05.

### Study approval.

All animal and human studies were conducted in accordance with the Declaration of Helsinki principles and approved by the Vanderbilt University Medical Center IACUC and the Vanderbilt University Medical Center IRB. The human retrospective cohort analysis was approved by the Vanderbilt University Medical Center IRB (no. 145579) under the category of exempt studies (minimal risk). This was a retrospective analysis of already existing data, and no new human data were generated, nor were any patients contacted. Human gastric tissue samples used for qRT-PCR were acquired from the Cooperative Human Tissue Network, and these analyses were approved by the Vanderbilt University Medical Center IRB (no. 210729) under the category of a nonhuman subject study.

## Author contributions

JMN designed the research study, conducted experiments, acquired data, analyzed and interpreted data, and wrote and edited the manuscript. MBP scored the histologic and IHC data and edited the manuscript. SCS designed the human retrospective cohort analysis, acquired, analyzed, and interpreted the human cohort analysis data, wrote the human cohort analysis sections, and edited the manuscript. JRG conducted animal breeding and genotyping, assisted with animal experiments, and edited the manuscript. JLH conducted DNA extractions for the microbiota experiments. CD conducted bioinformatic analysis for the microbiota experiments and wrote the microbiota methods. JDC conducted untargeted metabolomics experiments, analyzed the untargeted metabolomics data, and edited the manuscript. AGD performed IHC experiments. AEH and RAG analyzed and interpreted human cohort data. LEW conducted gastric organoid experiments, acquired and analyzed organoid data, and edited the manuscript. AS conducted and assisted with Western blot experiments. ABN conducted and assisted with *vacA* qRT-PCR analysis. MMA conducted and assisted with the Luminex chemokine and cytokine assays, provided reagents, and edited the manuscript. KTW assisted with the Luminex chemokine and cytokine data analyses, provided reagents, and edited the manuscript. MKW designed the human gastric TMA through the Cooperative Human Tissue Network. MWC assisted with untargeted metabolomics experiments, analyzed untargeted metabolomics data, wrote the untargeted metabolomics methods, and edited the manuscript. KLS assisted with untargeted metabolomics experiments and edited the manuscript. BPC provided wild-type C57BL/6 and *Tgr5^–/–^* mice and edited the manuscript. CRF conducted targeted bile acid experiments, acquired targeted bile acid data, and edited the manuscript. JPZ oversaw DNA extractions, 16S rRNA gene sequencing, bioinformatics analysis for the microbiota experiments, wrote the microbiota methods section, and edited the manuscript. RMP designed the research study, directed experiments, aided in data interpretation, and assisted with writing and editing of the manuscript.

## Supplementary Material

Supplemental data

Supplemental table 1

Supplemental table 2

## Figures and Tables

**Table 1:**
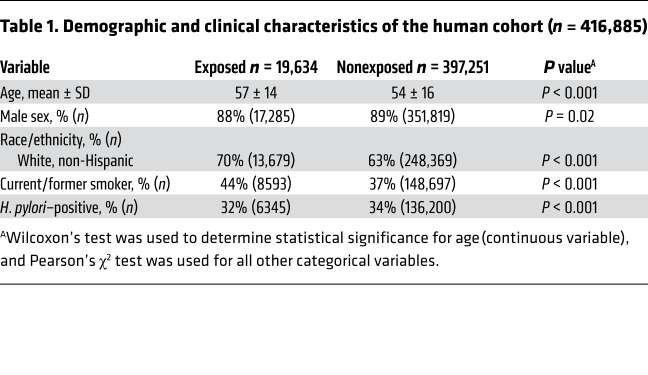
Demographic and clinical characteristics of the human cohort (*n* = 416,885)

**Table 2:**
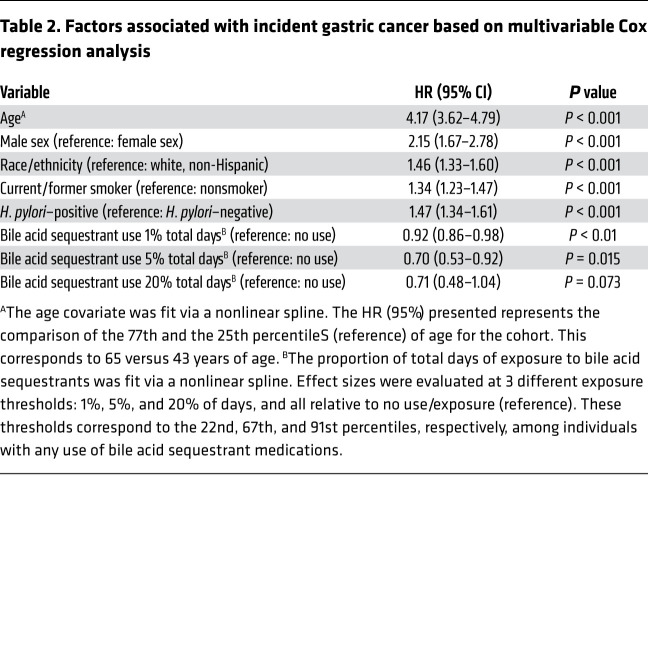
Factors associated with incident gastric cancer based on multivariable Cox regression analysis

**Table 3:**
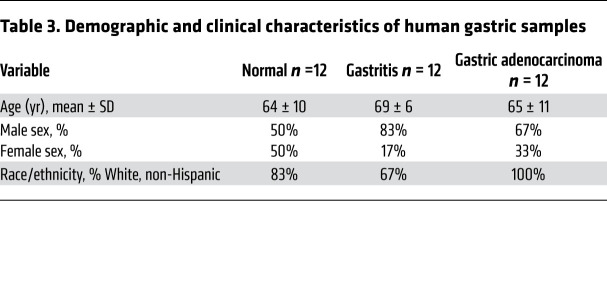
Demographic and clinical characteristics of human gastric samples

## Version 1 03/22/2022

In-Press Preview

## Version 2 05/16/2022

Electronic publication
