# Supplementary material for: Iron deficiency linked to altered bile acid metabolism promotes Helicobacter pylori–induced inflammation–driven gastric carcinogenesis
Source: J Clin Invest. 2022 May 16;132(10):e147822. doi: 10.1172/JCI147822 (PMC9106351; doi:10.1172/JCI147822)
Supplement: Supplemental data [file jci-132-147822-s049.pdf]

SUPPLEMENTAL METHODS

Complete blood counts (CBC). Blood was collected in tubes containing EDTA (Fisher) from mice maintained on either iron-replete or iron-depleted diets and CBC were performed. Parameters of iron deficiency were assessed and included hemoglobin, hematocrit, and mean corpuscular volume (MCV).

Ferritin ELISA. Blood was collected from mice and then sera were isolated. Levels of serum ferritin were determined by ELISA (Abcam), according to the manufacturer's instructions. Data were analyzed with Gen5 software (Synergy4, BioTek).

Quantitative *H. pylori* culture. Gastric tissue was harvested from mice and homogenized in sterile phosphate-buffered saline (PBS). Following serial dilution, samples were plated on selective trypticase soy agar plates with 5% sheep blood (Hemostat Lab) containing vancomycin (Sigma-Aldrich, 20 μg/ml), nalidixic acid (Sigma-Aldrich, 10 μg/ml), bacitracin (Calbiochem, 30 μg/ml), and amphotericin B (Sigma-Aldrich, 2 μg/ml) for isolation of *H. pylori*. Plates were incubated for five-seven days at 37°C with 5% CO_2. Colonies were identified as *H. pylori* based on characteristic morphology, Gram stain (Becton, Dickinson and Company), urease, and oxidase (Becton, Dickinson and Company) activities. Colonization density is expressed as log colony-forming units/gram of tissue. In vivo-adapted single colony isolates were harvested from infected mice maintained on iron-replete and iron-depleted diets for in vitro analyses.

Modified Steiner stain. Linear strips of gastric tissue, extending from the squamocolumnar junction through the proximal duodenum, were fixed in 10% neutral-buffered formalin (Azer Scientific), paraffin-embedded, and stained with a modified Steiner technique for the identification of *H. pylori* (1). A pathologist assessed *H. pylori* colonization density, which was scored 0-3 (negative (0), scarce (1), moderate (2), or abundant (3)) according to the updated Sydney System (2), in the antrum, transition zone, and corpus. Total colonization density was defined as the sum of colonization densities in the antrum, transition zone, and corpus. The topographical colonization

densities for the antrum, transition zone, or corpus are shown as a percentage of the total colonization density. Data are represented as average topographical *H. pylori* colonization density per mouse.

Mouse chemokine and cytokine multiplex bead array. Gastric linear strips extending from the squamocolumnar junction through the proximal duodenum were lysed in 200 μL of IP lysis buffer (ThermoFisher Scientific) containing protease and phosphatase inhibitors (Roche). Lysates were diluted 1:3 in assay buffer and mixed with magnetic beads according to the manufacturer's instructions (Millipore), as previously performed (3). Data were acquired and analyzed using the Millipore software platform. Data were expressed as pg of chemokine or cytokine/mg of gastric tissue.

Western blot analysis. AGS human gastric epithelial cells (ATCC, mycoplasma-negative) were grown in RPMI 1640 (Life Technologies) with 10% fetal bovine serum at 37°C with 5% CO_2. Wild-type *H. pylori* strain PMSS1, a PMSS1 *cagE*$^-$ isogenic mutant (negative bacterial control), and in vivo-adapted *H. pylori* strains were co-cultured with AGS cells at a MOI of 100:1 for six hours. For DCA experiments, co-cultures were also mock treated with vehicle control (PBS) or treated with 50 μM DCA (Sigma-Aldrich). Protein lysates were harvested, separated by SDS-PAGE, and transferred to PVDF membranes (Thermo Scientific). Levels of total CagA (anti-CagA antibody, Austral Biologicals, HPP-5003-9) and phosphorylated CagA (anti-pY99 antibody, Santa Cruz, sc-7020) as a measure of translocated CagA, were determined, as previously performed (4). Protein intensities were quantified using ImageJ 1.50i (National Institutes of Health).

Quantitative real-time PCR (qRT-PCR) for *H. pylori* gene expression. In vivo-adapted *H. pylori* strains were isolated from C57BL/6 and INS-GAS mice maintained on either iron-replete or iron-depleted diets. RNA was extracted from minimally passaged, log phase *H. pylori* strains and *vacA* gene expression was assessed by qRT-PCR. RNA was isolated using the RNeasy® RNA isolation kit (Qiagen), according to the manufacturer's instructions, and RNA was treated with RNase-free DNase (Promega). Reverse transcriptase PCR (Applied

Biosystems) and quantitative real-time PCR (Applied Biosystems, 7300 Real-Time PCR System) were performed, according to the manufacturer's instructions. Levels of *H. pylori vacA* mRNA expression (primers F: ACAACAAACACACCGCAAAA; R: CCTGAGACCGTTCCTACAGC) (5) were standardized to levels of *H. pylori 16S* mRNA expression (primers F: TGCGAAGTGGAGCCAATCTT; R: GGAACGTATTCACCGCAACA) using PowerTrack SYBR green master mix (ThermoFisher Scientific).

Microbial DNA extraction and 16S rRNA gene sequencing. Sample collection, preparation, and sequencing was performed as previously described (6). Briefly, linear strips of gastric tissue, extending from the squamocolumnar junction to the proximal duodenum were harvested and immediately frozen at −80°C. For analysis of the microbiota, gastric tissue samples were homogenized in 0.1 mm glass bead tubes with PowerBead solution (QIAGEN) and 50 mM TCEP solution (Thermo Scientific). Microbial genomic DNA was extracted using the PowerSoil DNA isolation kit (QIAGEN), according to the manufacturer's instructions. The V4 region of the 16S rRNA gene from each sample was amplified and sequenced using the Illumina Sequencing platform at the Children's Hospital of Philadelphia Microbiome Center. Sequence processing and analysis was performed using the QIIME2 pipeline. DADA2 was used for sequence quality filtering and denoising. Samples with less than 1000 assigned reads were excluded from further analysis. Student's t-test was used to determine statistical significance between treatment groups for Shannon diversity. Permutational multivariate analysis of variance (PERMANOVA) testing was used to determine statistical significance between the community structure (β-diversity) of different treatment groups based on weighted UniFrac distance matrices. aldex2 was used for differential taxa abundance analysis. Welch's t-test was used to determine the statistical significance in differential abundance, and the resulted *P* value was corrected by Benjamini-Hochberg method. Qiime2R package was used to generate the bar charts and PCoA plots. The code used for microbiome analyses is available on github and the raw sequence data has been deposited in the NCBI Sequence Read Archive (SRA, BioProject ID PRJNA811510).

Untargeted metabolomics. Gastric tissue samples were weighed and homogenized at 0°C by sonication (Fisher Sonic Dismembrator, 3 x 15 sec, power=3) to a final tissue density of 50 mg/mL in H_2O containing 50 mM NH_4OAc. Tissue debris was removed by centrifugation (1000 rpm, 10 minutes, 4°C); supernatants were then transferred to clean Eppendorf tubes and stored at -20°C until analysis. Tissue homogenates (100 µL) were thawed on ice and diluted with 100 µL of acetonitrile/water (1:1) containing an internal standard, carbamazepine (0.85 µM). Diluted homogenates were then centrifuged (1000 rpm, 10 minutes, 4°C), and supernatants were transferred to autosampler vials equipped with polypropylene small-volume inserts and Teflon-lined rubber septa. Samples were injected in duplicate using a Vanquish ultrahigh performance liquid chromatography (UHPLC) system interfaced to a Q Exactive HF quadrupole/orbitrap mass spectrometer (ThermoFisher Scientific). Chromatographic separation was performed with a reverse-phase Hypersil Gold-AQ C18 column (1.9 µm, 2.1x150mm, Thermo) at a flow rate of 300 µl/min. Mass spectra were acquired over a precursor ion scan range of *m/z* 70 to 1,050 at a resolving power of 60,000 using the following ESI source parameters: spray voltage 5 kV (3 kV in negative mode); capillary temperature 320°C; S-lens RF level 60 V; N_2 sheath gas 40; N_2 auxiliary gas 10; auxiliary gas temperature 100°C. MS/MS spectra were acquired for the top seven most abundant precursor ions with an MS/MS AGC target of 1e5, a maximum injection time of 50 ms, and a stepped normalized collision energy of 15,30,50. Chromatographic alignment, peak picking, and statistical comparisons were performed using Compound Discoverer v. 3.0 (ThermoFisher Scientific). All differential features (samples vs. controls) having a P value of <0.05 and a fold change of >1.5 were processed for molecular matches in the Chemspider, mzCloud, HMDB, and KEGG databases based on precursor ion exact masses (± 5 ppm) and MS/MS fragmentation patterns. Metabolite matches were then filtered to exclude biologically irrelevant drugs and environmental contaminants, and the finalized list of putative identifications were mapped to relevant biological pathways using the Metabolika software module.

Bile acid targeted analysis. All bile acids were purchased from C.D.N. Isotopes Inc. (Pointe Claire, Montreal, PQ, Canada). Tauro-β-muricholic-d4 acid (5β-cholanic acid-3α,6β,7β-triol-N-[2-sulphoethyl]-amide-2,2,4,4-d4,

TβMCA-d4) was purchased from US Biological Corporation (Swampscott, MA). High-performance liquid chromatography-grade water, acetonitrile, methanol, ammonium acetate, and ammonia were purchased from Sigma Chemicals (St Louis, MO). Formic acid was purchased from Thermo Scientific. Stock solutions of 2.5 mM of all bile acids (THCA, HCA, TαMCA, TβMCA, TωMCA, HDCA, THDCA: 10 mM) were used to prepare calibrators with concentrations of 100 μM in methanol. For the preparation of calibrators, bile acids were mixed to achieve final concentrations of 20, 2.5, 0.75, 0.25, 0.05, 0.015, and 0.005 μM, respectively. To prepare 20 mL of a 2.0 nM internal standard, 250 μL each of d4-CDCA, d4-TCA, and d4-GCDCA, and 500 μL d4-CA, d4-TβMCA, and d4-GCA were added to 20% (vol/vol) acetonitrile. For bile acid measurements, the following were added to 50 μL of homogenate: 200 μL of 100 mM aqueous sodium hydroxide and 50 μL of a 2-nM internal standard mix. The sample was then heated at 64°C for 30 minutes, centrifuged for 10 minutes at 14,400*g*, and the supernatant acidified to pH 7.0 with 50 μL of 0.1M hydrochloric acid. The sample was brought to a final volume of 1 mL with water and applied to a 1-mL (30 mg) Oasis HLB cartridge (Waters, Milford, MA) previously equilibrated first with 1 mL methanol, and then 1 mL water (7). The column-bound bile acids were washed with 1 mL 5% (vol/vol) aqueous methanol, followed by 1 mL 2% (vol/vol) aqueous formic acid. Bile acids were eluted from the column with 1 mL 2% (vol/vol) ammonia in methanol and the eluent evaporated to dryness using a rotary evaporator at 30°C for 2 hours. Samples were resuspended in 100 μL 25% (vol/vol) acetonitrile in water.

Mass spectrometry analysis was performed using a TSQ Quantum mass spectrometer (ThermoFinnigan, Sunnyvale, CA) equipped with an ESI probe in negative-ion mode. The following (optimized) parameters were used for the detection of the analytes and the internal standard: N2 sheath gas, 49 psi; N2 auxiliary gas, 25 psi; spray voltage, 3.0 kV; source collision-induced dissociation, 25 V; capillary temperature, 300°C; capillary offset, −35 V; tube lens voltage, 160 V; Q2 gas pressure, 1.5 mtor; Q3 scan width 1 m/z; Q1/Q3 peak widths at half-maximum, 0.7 m/z. Calibration curves and concentrations of individual bile acids were calculated by LCQuan 2.5.5 software (ThermoFinnigan). Concentrations of individual bile acids were calculated from peak area in the chromatogram detected with selection reaction monitoring relative to the appropriate internal standard.

An Acquity ultra performance liquid chromatography system (Waters, Milford, MA) was used with an Acquity ultra performance liquid chromatography BEH C18 1.7-µm, 2.1 × 100-mm column (Waters), and heated to 50°C, and a binary solvent system of 20% (vol/vol) acetonitrile in water (mobile phase A) and 80% (vol/vol) acetonitrile in water (mobile phase B), both containing 1 mM ammonium acetate, were used to resolve plasma bile acids as previously described (8). Mass spectrometry analysis was performed using a TSQ Quantum mass spectrometer (ThermoFinnigan) equipped with an ESI probe in negative-ion mode. The following (optimized) parameters were used for the detection of the analytes and the internal standard: N2 sheath gas, 49 psi; N2 auxiliary gas, 25 psi; spray voltage, 3.0 kV; source CID, 25 V; capillary temperature, 300°C; capillary offset, −35 V; tube lens voltage, 160 V; Q2 gas pressure, 1.5 mtor; Q3 scan width 1 m/z; Q1/Q3 peak widths at half-maximum, 0.7 m/z. Calibration curves and concentrations of individual bile acids were calculated by LCQuan 2.5.5 software (ThermoFinnigan). Concentrations of individual bile acids were calculated from peak area in the chromatogram detected with SRM relative to the appropriate internal standard.

Human retrospective cohort analysis. The Corporate Data Warehouse contains longitudinal electronic medical record data for all veterans who obtain their care through the Veterans Health Administration (VHA). These data undergo routine quality checks and were reliable starting in the year 2000. We identified over 15 million veterans nationally with established care through the VHA between January 1, 2000 and May 31, 2020. From this initial cohort, we identified all individuals who had undergone serological or non-serological laboratory testing for *H. pylori* and had results available. In addition to lack of *H. pylori* testing, other exclusion criteria included age <18 years or >95 years old, or any cancer diagnosis (except non-melanoma skin cancer) prior to study entry. The study period was defined as January 1, 2005 (to ensure at least five years of data capture would be available) to May 31, 2020. Individuals with negative follow-up time (data errors) or less than 30 days of follow-up time following study entry were also excluded. The analytic cohort after inclusion/exclusion criteria were applied comprised 416,885 individuals. The following data were abstracted from the medical record: age at study entry, sex, race/ethnicity, smoking status (ever smoker versus never smoker), and *H. pylori* status (positive versus negative).

Pharmacy data were queried for filled prescriptions of bile acid sequestrants, which included cholestyramine, colestipol, and colesevelam. In the electronic medical record system used for this cohort analysis, providers are not prompted to enter an associated diagnosis when prescribing medications. As such, although all patients included in this analysis had linkage to pharmacy data and details of filled prescriptions, there was no information on the indication for bile acid sequestrant use. The primary outcome, gastric cancer, was determined based on International Classification of Diseases (ICD)-9 [malignant neoplasm of the stomach: 151*] and ICD-10 [C16*] codes, and separated based on anatomic classification as cardia (151.0 or C16.0) versus noncardia/not otherwise specified/other (151.1-151.9 or C16.1-C16.9).

Human gastric organoids. Primary human gastric organoids were generated as previously described (9). Briefly, gastric tissue was washed, digested, and isolated glands were incubated in Geltrex® (ThermoFisher) to form primary 3D gastric organoids (10). To convert primary 3D gastric organoids to primary 2D monolayers, Geltrex® was removed and 3D gastric organoids were plated on Geltrex®-coated plates. Primary 2D gastric monolayers were then co-cultured with or without *H. pylori* strain PMSS1 at a multiplicity of infection (MOI) of 10:1. Following bacterial adherence, monolayers were treated with or without 100 μM DCA (Sigma-Aldrich) and *H. pylori*-monolayer co-cultures were harvested 1, 4, and 24 hours post-challenge. Gastric organoid co-cultures were assessed for p-EGFR (anti-p-EGFR, Biocare Medical, 50-831-75) by immunofluorescence (IF), IL-8 secretion by ELISA (R&D Systems), and proliferation via EdU (Invitrogen). For immunofluorescence analyses, cells were fixed and permeabilized with Triton X-100 (Promega), and blocked with Dako Protein Block Solution (Agilent). Samples were incubated with anti-p-EGFR overnight at 4°C before detection with Alexa Fluor secondary antibody (Invitrogen A21206). Nuclei were detected using Hoescht (Invitrogen). Images were acquired on an Olympus FV-1000 confocal microscope. All animal studies were conducted in accordance with the Vanderbilt University Medical Center Institutional Animal Care and Use Committee (IACUC).

Quantitative PCR (qRT-PCR) for *TGR5* in human tissues. Snap frozen de-identified human gastric tissue samples were acquired from the Cooperative Human Tissue Network (CHTN). These analyses were approved under IRB #210729 under the category of a non-human subject study. Normal gastric tissue (*N*=12), gastric tissues with gastritis alone (*N*=12), and gastric tissues with gastric adenocarcinoma (*N*=12) were disrupted and homogenized using ZR BashingBead Lysis tubes (Zymo Research). RNA was then isolated using the RNeasy® RNA isolation kit (Qiagen), according to the manufacturer's instructions, and RNA was treated with RNase-free DNase (Promega). Reverse transcriptase PCR (Applied Biosystems) and quantitative real-time PCR (Applied Biosystems, 7300 Real-Time PCR System) were performed, according to the manufacturer's instructions. Levels of *TGR5* mRNA expression were standardized to levels of *GAPDH* mRNA expression using TaqMan gene expression assays (ThermoFisher Scientific).

Murine model of *Tgr5*-deficiency. Male and female wild-type C57BL/6J, heterozygous *Tgr5*$^{+/-}$, and homozygous *Tgr5*$^{-/-}$ mice were obtained from Bethany Cummings at Cornell University (11). Mice were bred and housed in the Vanderbilt University Medical Center animal care facilities in a room with a 12-hour light-dark cycle at 21°C to 22°C. Wild-type C57BL/6J WT and *Tgr5*$^{-/-}$ littermates were maintained on an iron-replete standard rodent diet (LabDiet, PicoLab® Laboratory Rodent Diet, 5L0D*) and then orogastrically challenged with Brucella broth or *H. pylori* strain PMSS1. Mice were euthanized eight weeks post-challenge (Supplementary Figure 1F). All animal studies were conducted in accordance with the Vanderbilt University Medical Center Institutional Animal Care and Use Committee (IACUC).

SUPPLEMENTAL REFERENCES

1. Garvey W, Fathi A, Bigelow F, and Wynnchuk M. Revised modified Steiner to enhance visability of spirochetes. *J Histotechnol*. 1995;18(1):57-60.
2. Dixon MF, Genta RM, Yardley JH, and Correa P. Classification and grading of gastritis. The updated Sydney System. International workshop on the histopathology of gastritis, Houston 1994. *Am J Surg Pathol*. 1996;20(10):1161-81.
3. Krakowiak MS, Noto JM, Piazuelo MB, Hardbower DM, Romero-Gallo J, Delgado A, et al. Matrix metalloproteinase 7 restrains *Helicobacter pylori*-induced gastric inflammation and premalignant lesions in the stomach by altering macrophage polarization. *Oncogene*. 2015;34(14):1865-71.
4. Noto JM, Gaddy JA, Lee JY, Piazuelo MB, Friedman DB, Colvin DC, et al. Iron deficiency accelerates *Helicobacter pylori*-induced carcinogenesis in rodents and humans. *J Clin Invest*. 2013;123(1):479-92.
5. Caston RR, Loh JT, Voss BJ, McDonald WH, Scholz MB, McClain MS, et al. Effect of environmental salt concentration on the *Helicobacter pylori* exoproteome. *J Proteomics*. 2019;202:103374.
6. Noto JM, Zackular JP, Varga MG, Delgado A, Romero-Gallo J, Scholz MB, et al. Modification of the gastric mucosal microbiota by a strain-specific *Helicobacter pylori* oncoprotein and carcinogenic histologic phenotype. *MBio*. 2019;10(3).
7. Rodrigues CM, and Setchell KD. Performance characteristics of reversed-phase bonded silica cartridges for serum bile acid extraction. *Biomed Chromatogr*. 1996;10(1):1-5.
8. Albaugh VL, Banan B, Antoun J, Xiong Y, Guo Y, Ping J, et al. Role of bile acids and GLP-1 in mediating the metabolic improvements of bariatric surgery. *Gastroenterology*. 2019;156(4):1041-51 e4.
9. Teal E, Bertaux-Skeirik N, Chakrabarti J, Holokai L, and Zavros Y. Establishment of human- and mouse-derived gastric primary epithelial cell monolayers from organoids. *Methods Mol Biol*. 2018;1817:145-55.
10. Bartfeld S, and Clevers H. Organoids as model for infectious diseases: Culture of human and murine stomach organoids and microinjection of *Helicobacter pylori*. *J Vis Exp*. 2015(105).

11. McGavigan AK, Garibay D, Henseler ZM, Chen J, Bettaieb A, Haj FG, et al. TGR5 contributes to glucoregulatory improvements after vertical sleeve gastrectomy in mice. *Gut*. 2017;66(2):226-34.

SUPPLEMENTAL FIGURES

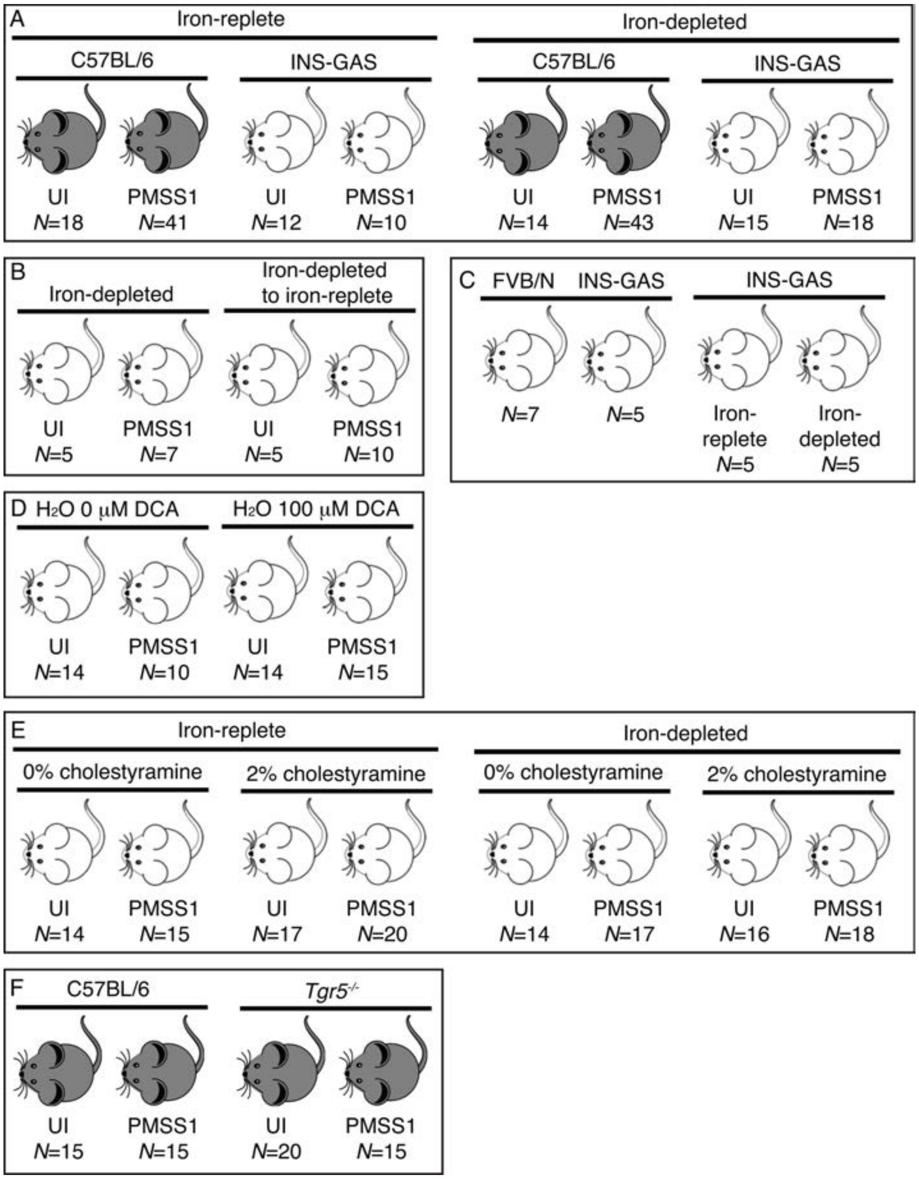

Supplemental Figure 1. Experimental design. (**A**) C57BL/6 mice (black) and male INS-GAS mice (white) were maintained on iron-replete or iron-depleted customized diets for two weeks prior to challenge and throughout the course of the experiment. Mice were challenged with Brucella broth, as an uninfected (UI), negative control or with wild-type *cag*-positive *H. pylori* strain PMSS1. Mice were euthanized eight weeks post-challenge. (**B**) Two groups of male INS-GAS mice were maintained on iron-depleted diets for two weeks prior to challenge with or without *H. pylori* strain PMSS1. One group was continued on an iron-depleted diet, while the other group was switched to an iron-replete diet two weeks post-challenge. Mice were euthanized eight weeks post-challenge. (**C**) The microbiota was assessed in uninfected male wild-type FVB/N mice versus INS-GAS mice as well as uninfected male INS-GAS mice maintained on iron-replete versus iron-depleted diets. (**D**) Male INS-GAS mice were maintained on an iron-replete standard rodent diet, challenged with Brucella broth (UI) or *H. pylori* strain PMSS1, and then provided water supplemented either with or without 100 μM deoxycholic acid (DCA) two weeks later. Mice were euthanized six weeks post-challenge. (**E**) Male INS-GAS mice were maintained on iron-replete or iron-depleted customized diets supplemented either with or without 2% cholestyramine for two weeks prior to challenge and throughout the course of the experiment. Mice were then challenged with Brucella broth (UI) or *H. pylori* strain PMSS1. Mice were euthanized eight weeks post-challenge. (**F**) Wild-type C57BL/6J and homozygous *Tgr5*$^{-/-}$ mice were maintained on an iron-replete standard rodent diet and then orogastrically challenged with Brucella broth (UI) or with *H. pylori* strain PMSS1. Mice were euthanized eight weeks post-challenge.

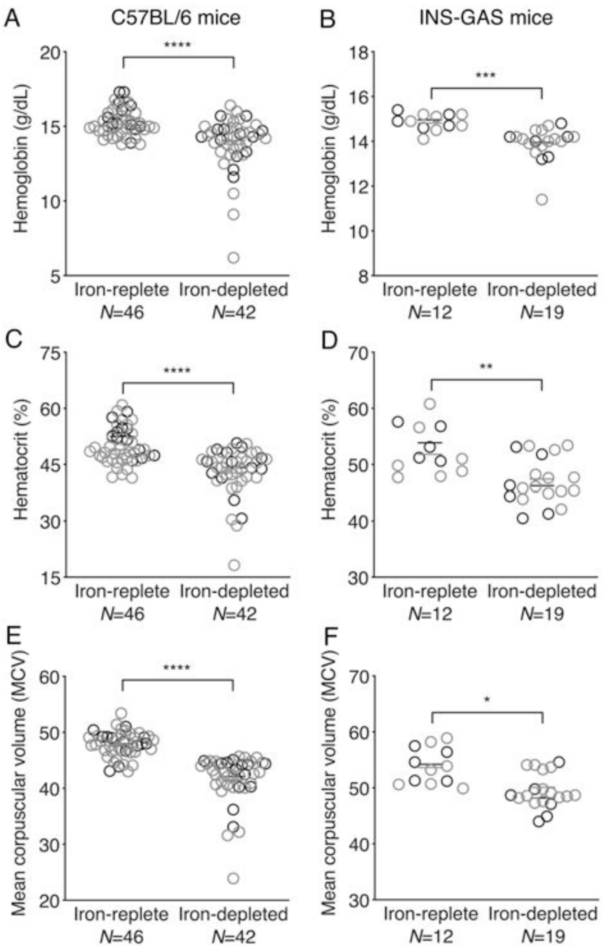

Supplemental Figure 2. Iron-depleted diets induce iron deficiency in both C57BL/6 and INS-GAS mice. Wild-type C57BL/6 mice (left panels) and transgenic hypergastrinemic INS-GAS mice (right panels) were maintained on iron-replete or iron-depleted diets for two weeks prior to challenge and throughout the course of the experiment. Mice were challenged with Brucella broth (UI) or wild-type *H. pylori* strain PMSS1 and were euthanized eight weeks post-challenge. Blood was harvested for CBC analysis from a subset of uninfected (black symbols) and *H. pylori*-infected (gray symbols) mice. Hemoglobin (**A-B**), hematocrit (**C-D**), and mean corpuscular volume (**E-F**) were assessed as parameters of iron deficiency in uninfected and *H. pylori*-infected C57BL/6 (*N*=88) and INS-GAS (*N*=31) mice from three independent experiments. Each point represents data from an individual animal and mean values are shown in scatter dot plots. Unpaired parametric t-test was used to determine statistical significance. ****, $P<0.0001$; ***, $P<0.001$; **, $P<0.01$; *, $P<0.05$

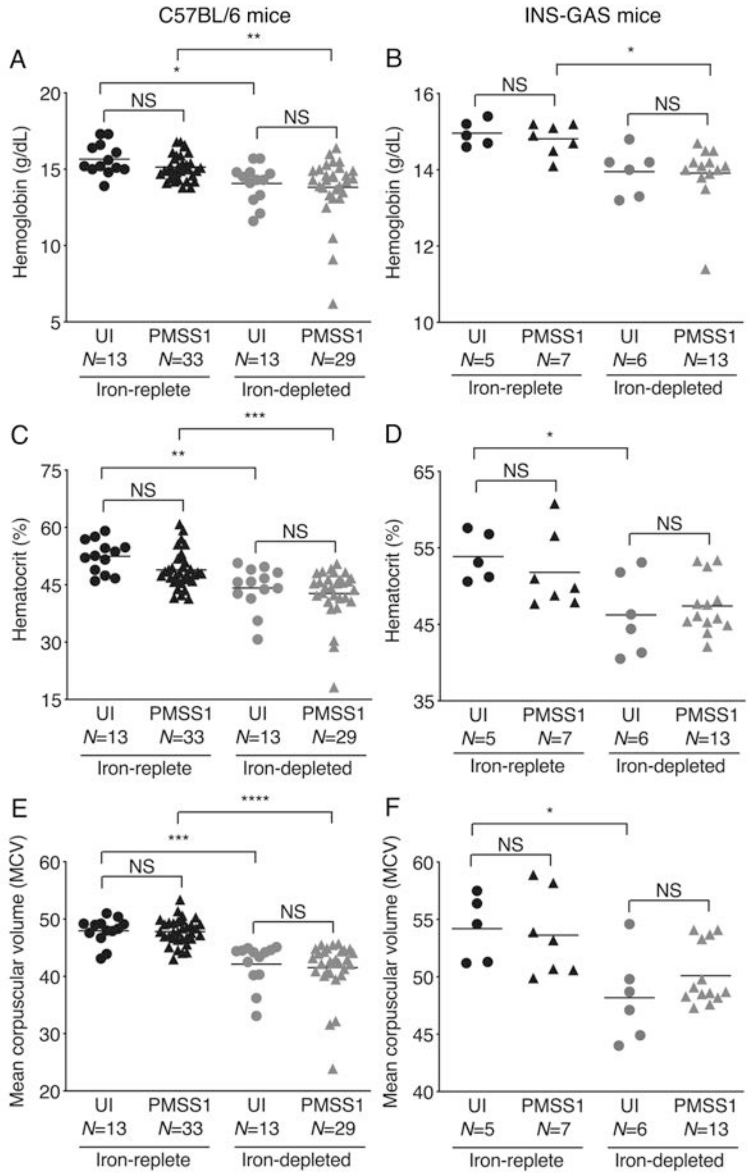

Supplemental Figure 3. Iron-depleted diets induce iron deficiency in both C57BL/6 and INS-GAS mice, but infection with *H. pylori* does induce iron deficiency or exacerbate iron deficiency. Wild-type C57BL/6 mice (left panels) and transgenic hypergastrinemic INS-GAS mice (right panels) were maintained on iron-replete or iron-depleted diets for two weeks prior to challenge and throughout the course of the experiment. Mice were challenged with Brucella broth (UI) or wild-type *H. pylori* strain PMSS1 and were euthanized eight weeks post-challenge. Blood was harvested for CBC analysis from a subset of uninfected and *H. pylori*-infected mice. Hemoglobin (**A-B**), hematocrit (**C-D**), and mean corpuscular volume (**E-F**) were assessed as parameters of iron deficiency in uninfected and *H. pylori*-infected C57BL/6 (*N*=88) and INS-GAS (*N*=31) mice from three independent experiments. Each point represents data from an individual animal and mean values are shown in scatter dot plots. One-way ordinary ANOVA with Sidak's multiple comparison test was used to determine statistical significance. ****, *P*<0.0001; ***, *P*<0.001; **, *P*<0.01; *, *P*<0.05; NS, not statistically significant.

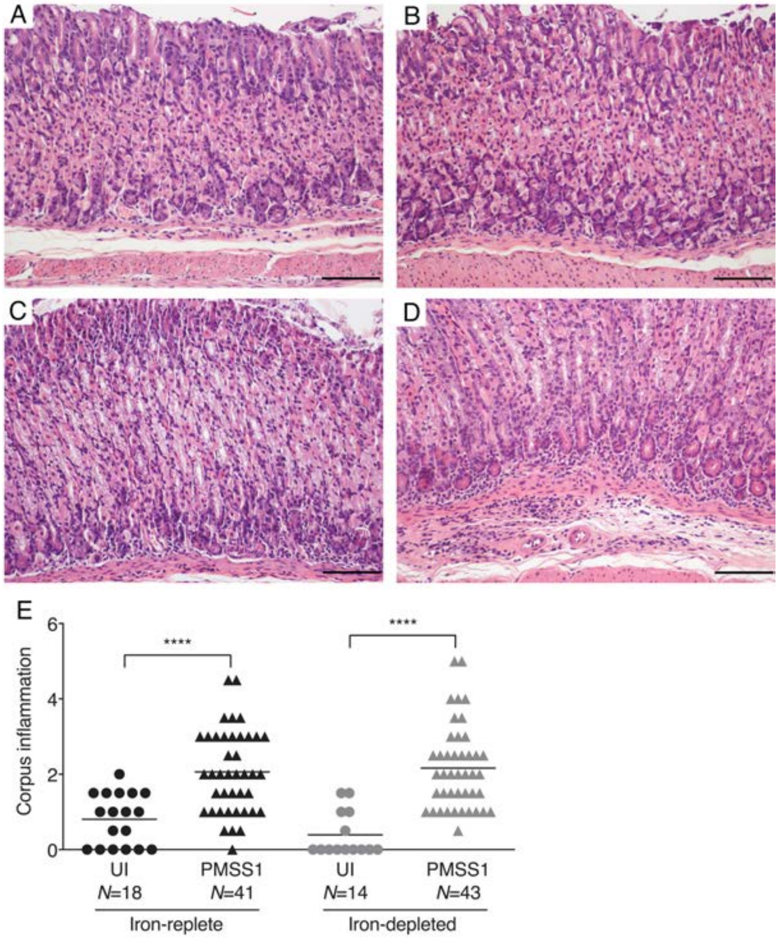

Supplemental Figure 4. *H. pylori*-induced gastric inflammation in the corpus of C57BL/6 mice. Wild-type male and female C57BL/6 mice were maintained on iron-replete (*N*=59) or iron-depleted (*N*=57) diets and then challenged with Brucella broth (UI) or *H. pylori* strain PMSS1. Mice were euthanized eight weeks post-challenge. Gastric tissue was fixed, paraffin-embedded, and stained with hematoxylin and eosin. Representative images of histology from the corpus of uninfected mice maintained on iron-replete (**A**) or iron-depleted (**B**) diets and representative images from *H. pylori*-infected mice maintained on iron-replete (**C**) or iron-depleted (**D**) diets are shown (200X). Scale bars represent 100 microns. (**E**) Levels of total gastric inflammation (0-6) were assessed in the corpus. Each point represents data from an individual animal from three independent experiments and mean values are shown in scatter dot plots. One-way ordinary ANOVA with Sidak's multiple comparison test was used to determine statistical significance. Only statistically significant comparisons are denoted. ****, $P<0.0001$

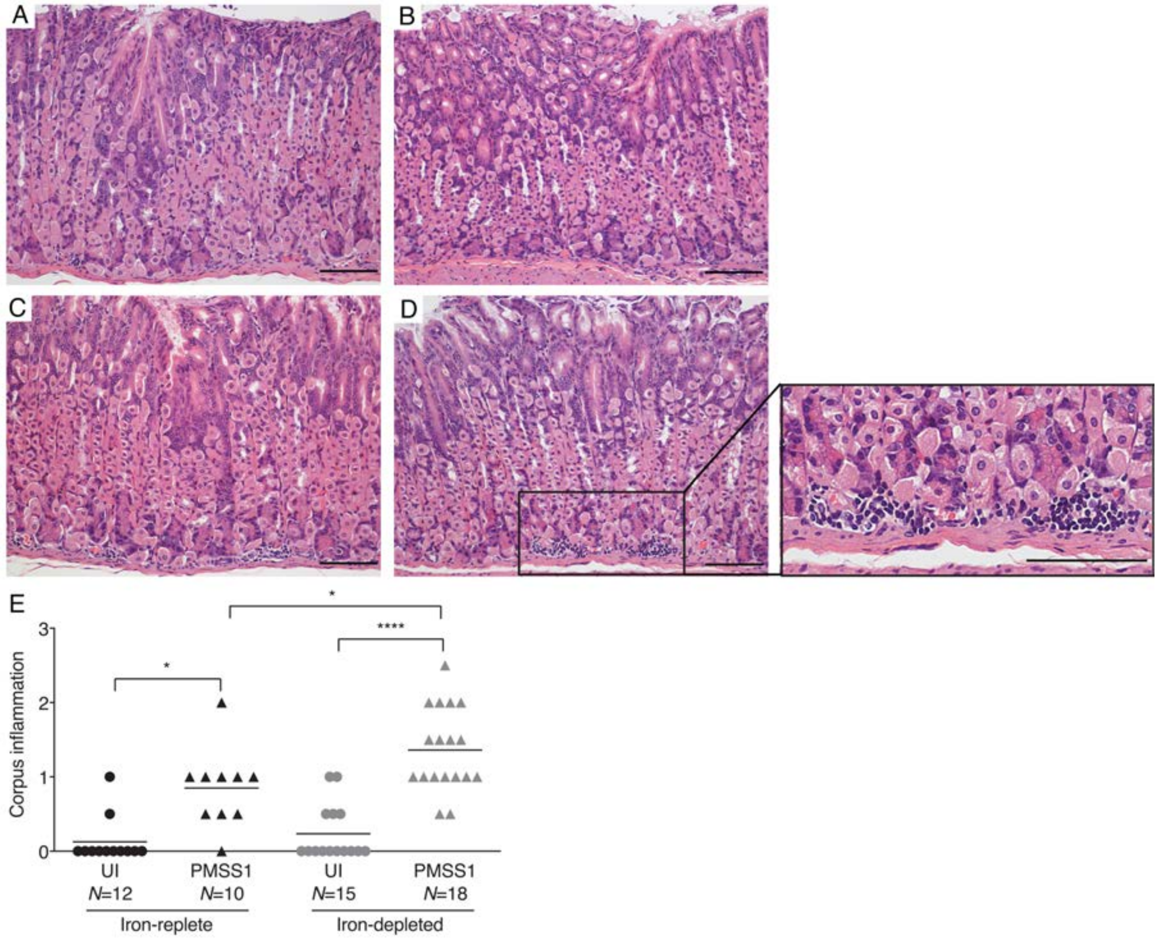

Supplemental Figure 5. Iron deficiency augments *H. pylori*-induced gastric inflammation and injury in the corpus of INS-GAS mice. Male INS-GAS mice were maintained on iron-replete (*N*=22) or iron-depleted (*N*=33) diets and then challenged with Brucella broth (UI) or *H. pylori* strain PMSS1. Mice were euthanized eight weeks post-challenge. Gastric tissue was fixed, paraffin-embedded, and stained with hematoxylin and eosin. Representative images of histology from the corpus of uninfected mice maintained on iron-replete (**A**) or iron-depleted (**B**) diets and representative images from *H. pylori*-infected mice maintained on iron-replete (**C**) or iron-depleted (**D**) diets are shown (200X and 400X). Scale bars represent 100 microns. (**E**) Levels of total gastric inflammation (0-6) were assessed in the corpus. Each point represents data from an individual animal from three independent experiments and mean values are shown in scatter dot plots. One-way ordinary ANOVA with Sidak's multiple comparison test was used to determine statistical significance. Only statistically significant comparisons are denoted. ****, $P<0.0001$; *, $P<0.05$

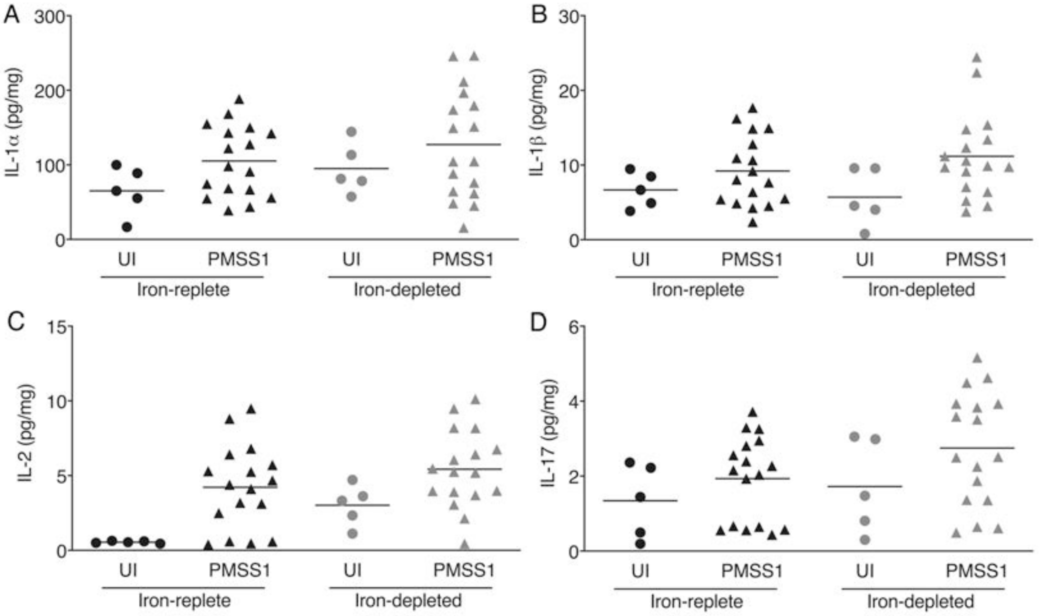

Supplemental Figure 6. Levels of chemokines and cytokines among *H. pylori*-infected C57BL/6 mice. C57BL/6 mice were maintained on iron-replete or iron-depleted diets and then challenged with Brucella broth (UI) or wild-type *H. pylori* strain PMSS1. Mice were euthanized eight weeks post-challenge. Gastric tissue was analyzed using a cytokine/chemokine multiplex bead array. Data were acquired and analyzed using the Millipore software platform and expressed as pg of chemokine per mg of gastric tissue. Levels of IL-1α (**A**), IL-1β (**B**), IL-2 (**C**), and IL-17 (**D**) were increased by *H. pylori* infection, but were not significantly changed under conditions of iron deficiency. Each point represents data from an individual animal from three independent experiments. C57BL/6 mice: iron-replete UI (*N*=5) and PMSS1 (*N*=17), iron-depleted UI (*N*=5) and PMSS1 (*N*=17). Mean values are shown in scatter dot plots. One-way ordinary ANOVA with Sidak's multiple comparison test was used to determine statistical significance. None of the comparisons were statistically significant.

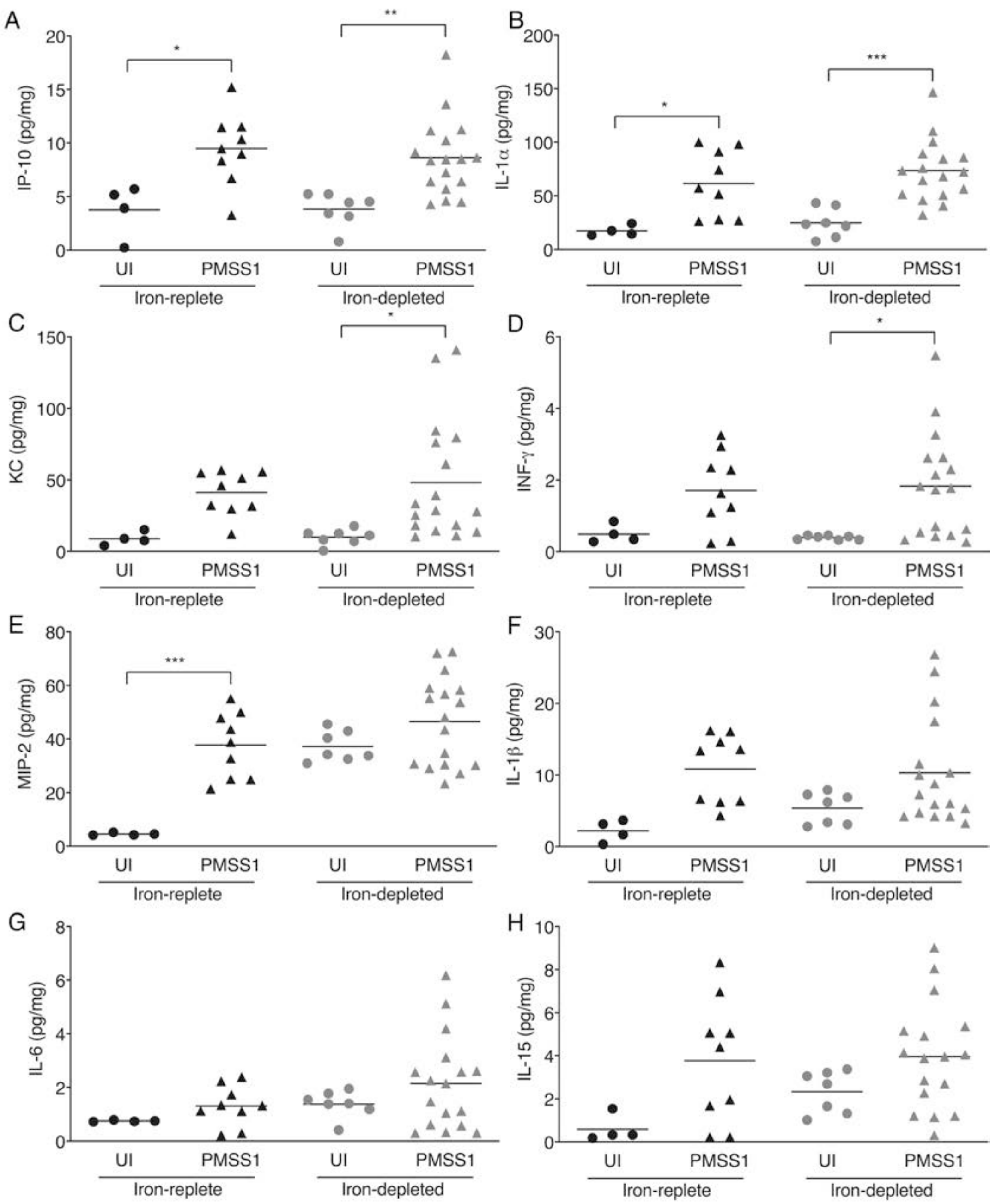

Supplemental Figure 7. Levels of chemokines and cytokines among *H. pylori*-infected INS-GAS mice. Male INS-GAS mice were maintained on iron-replete or iron-depleted diets and then challenged with Brucella broth (UI) or wild-type *H. pylori* strain PMSS1. Mice were euthanized eight weeks post-challenge. Gastric tissue was homogenized, lysed, and analyzed using a cytokine/chemokine multiplex bead array. Data were acquired and analyzed using the Millipore software platform and expressed as pg of chemokine per mg of gastric tissue. Levels of IP-10 (**A**), IL-α (**B**), KC (**C**), IFN-γ (**D**), MIP-2 (**E**), IL-1β (**F**), IL-6 (**G**), and IL-15 (**H**) were increased by *H. pylori*, but were not significantly changed under condition of iron deficiency. Each point represents data from an individual animal from two independent experiments. INS-GAS mice: iron-replete UI (*N*=4) and PMSS1 (*N*=9), iron-depleted UI (*N*=7) and PMSS1 (*N*=17). Mean values are shown in scatter dot plots. One-way ordinary ANOVA with Sidak's multiple comparison test (**A-H**) was used to determine statistical significance. Only statistically significant comparisons are denoted. ***, $P<0.001$; **, $P<0.01$; *, $P<0.05$

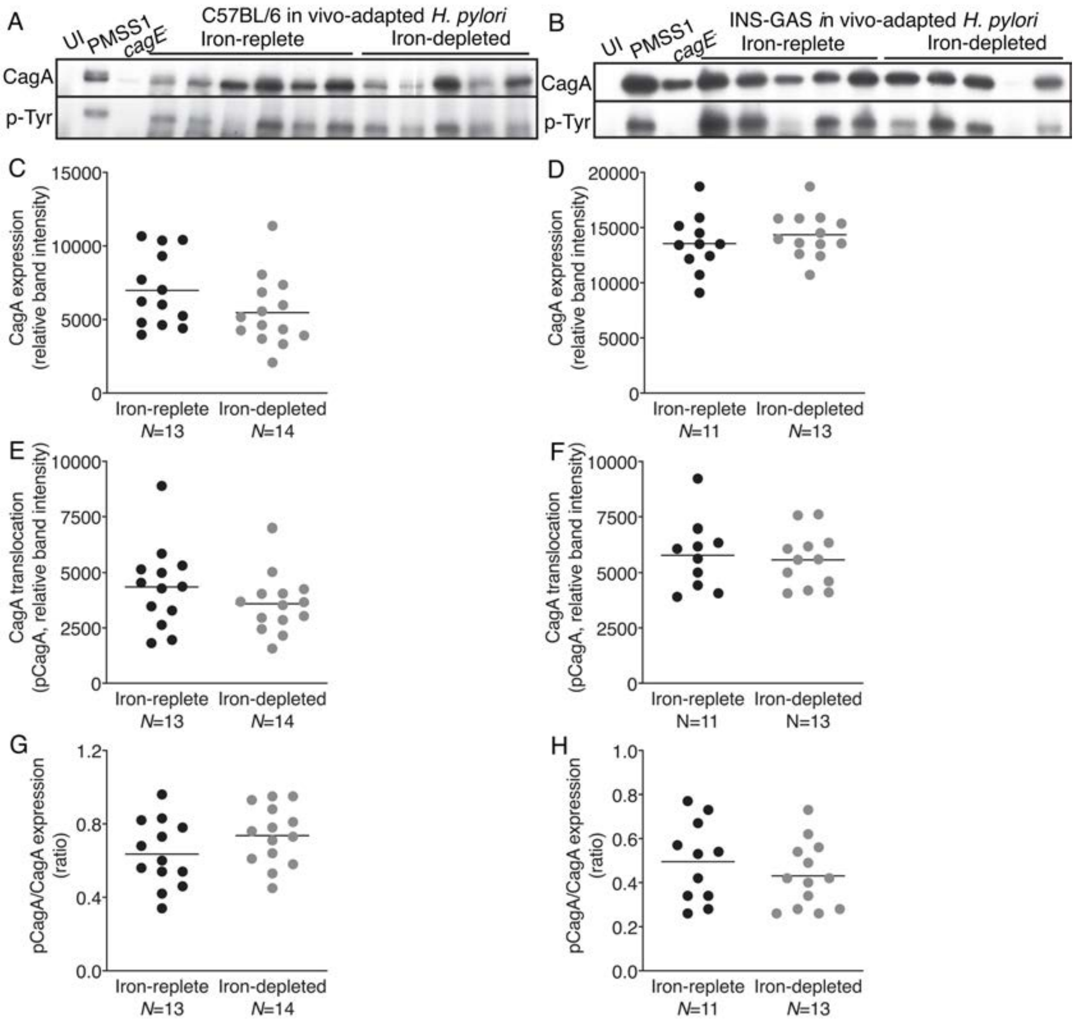

Supplemental Figure 8. In vivo-adaptation of *H. pylori* does not alter *cag* type IV secretion system function. C57BL/6 (left panels) and INS-GAS (right panels) mice were maintained on iron-replete or iron-depleted diets and then challenged with Brucella broth (UI) or wild-type *H. pylori* strain PMSS1. Mice were euthanized eight weeks post-challenge and gastric tissue was harvested for isolation of *H. pylori* output strains adapted to iron-replete or iron-depleted conditions. Wild-type *H. pylori* strain PMSS1, a PMSS1 *cagE* isogenic mutant, and in vivo-adapted *H. pylori* strains were co-cultured with AGS gastric epithelial cells for six hours and protein lysates were harvested for Western blot analysis. Levels of total CagA (**A-D**), phosphorylated CagA (p-Tyrosine, **A-B**, **E-F**), and the ratio of phosphorylated CagA (pCagA) to total CagA was also determined (G-H) from three independent experiments. Each point represents data from an individual *H. pylori* strain. Mean values are shown in scatter dot plots. Unpaired parametric t-test was used to determine statistical significance and none of the comparisons were statistically significant.

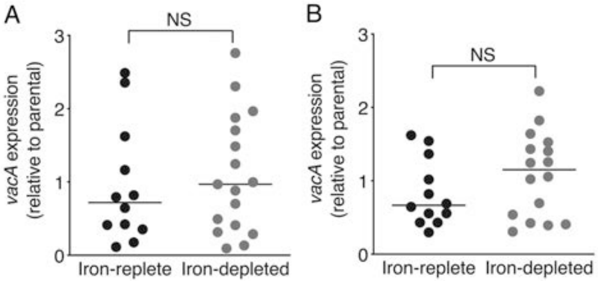

Supplemental Figure 9. In vivo-adaptation of *H. pylori* does not alter *vacA* gene expression. RNA was extracted from minimally passaged *H. pylori* strains harvested from C57BL/6 (*N*=29, **A**) or INS-GAS (*N*=28, **B**) mice maintained on either iron-replete or iron-depleted diets and qRT-PCR was performed to assess *vacA* gene expression. Gene expression levels were standardized to levels of *16S* gene expression and are shown as fold relative to the parental strain PMSS1 (2^-(ΔΔCT)) from three independent experiments. Each point represents data from an individual *H. pylori* strain and mean values are shown in scatter dot plots. Unpaired parametric t-test was used to determine statistical significance and none of the comparisons were statistically significant. NS, not statistically significant.

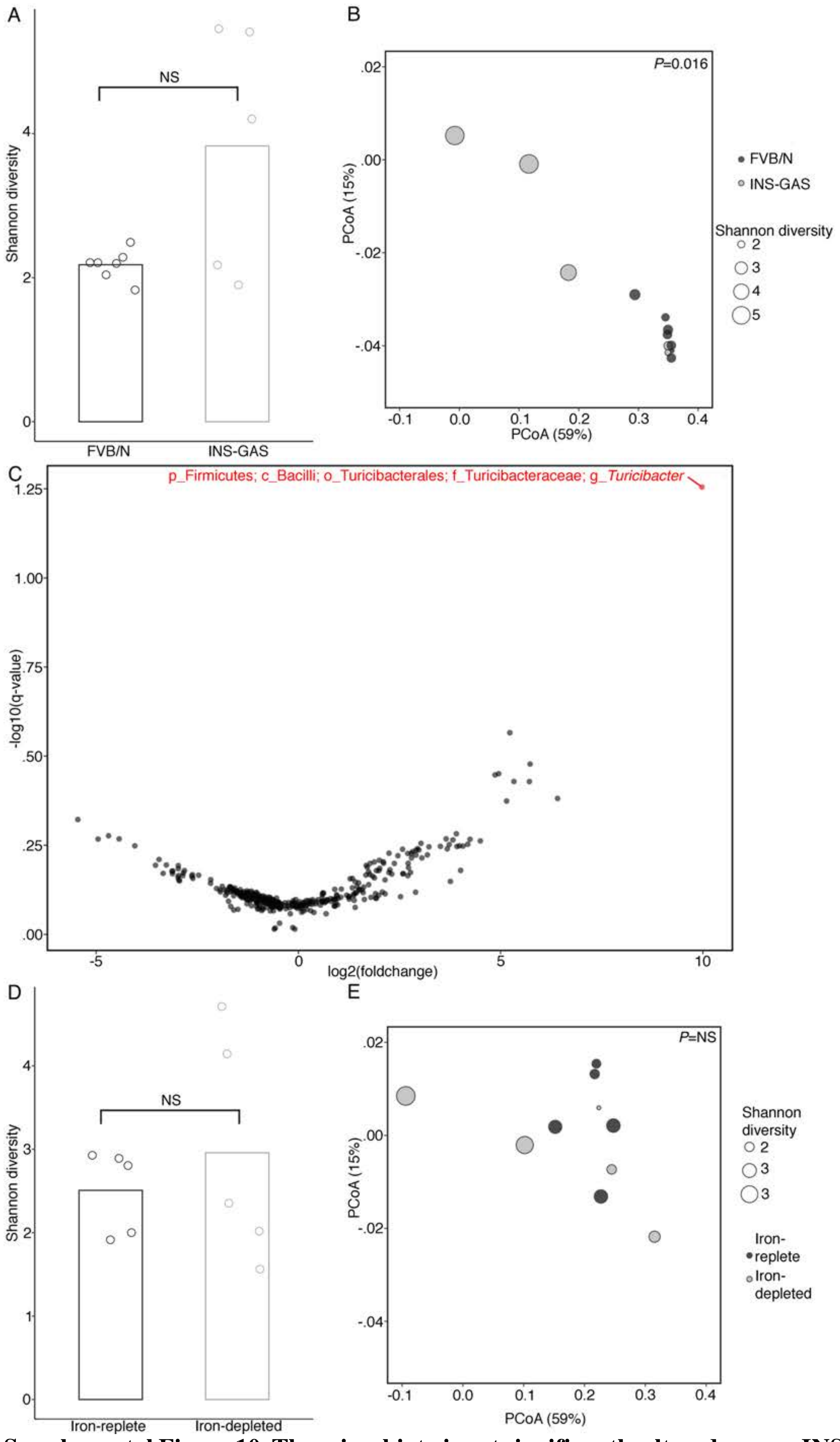

Supplemental Figure 10. The microbiota is not significantly altered among INS-GAS mice under conditions of iron deficiency. Gastric tissue was harvested from uninfected wild-type FVB/N (*N*=7) and INS-GAS (*N*=5)

male mice (**A-C**) and from uninfected male INS-GAS mice maintained on iron-replete (*N*=5) or iron-depleted (*N*=5) diets (**D-E**) for 16S rRNA gene sequencing and microbiota analyses. Shannon diversity (**A**, **D**), β-diversity (**B**, **E**), and differential abundance (**C**) are shown. PCoA plot was generated using weighted UniFrac distances. Each point represents data from an individual animal and percent variance is shown for each PCoA axis. Student's t-test was used to determine statistical significance between treatment groups for Shannon diversity (**A**, **D**). PERMANOVA was used to determine statistical significance in β-diversity based on weighted UniFrac distance matrices (**B**, **E**). Welch's t-test was used to determine statistical significance between treatment groups for differential abundance (**C**) and statistically significant taxa are shown in red. NS, not statistically significant.

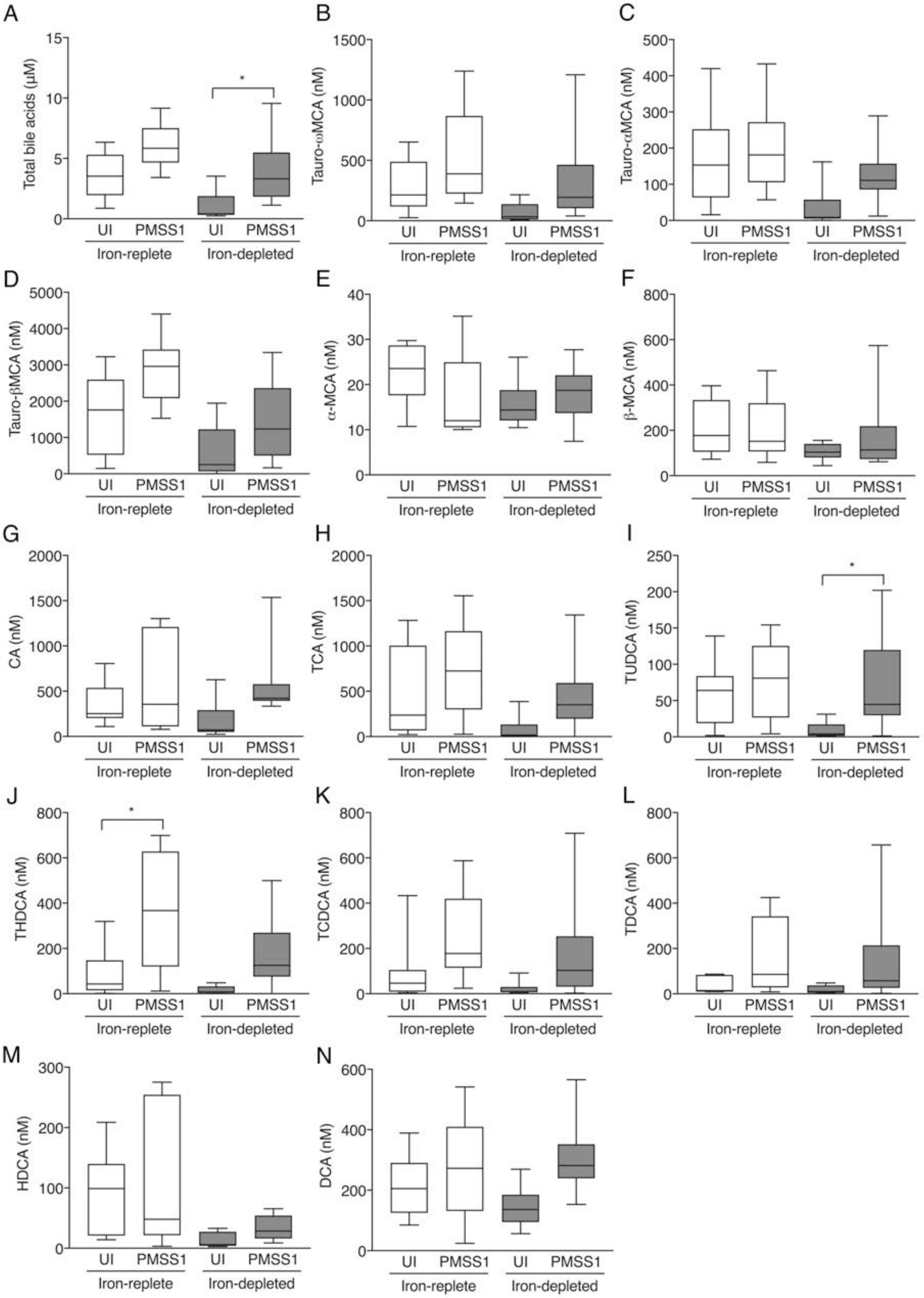

Supplemental Figure 11. Levels of bile acids among *H. pylori*-infected C57BL/6 mice. C57BL/6 mice were maintained on iron-replete or iron-depleted diets and then challenged with Brucella broth (UI) or wild-type *H. pylori* strain PMSS1. Mice were euthanized eight weeks post-challenge. Gastric tissue was processed for targeted bile acid analyses. Levels of total bile acids (**A**) muricholic acid (**B-F**), cholic acid (**G-H**), and deoxycholic acid (**I-N**) are shown. Ten mice were analyzed per group from two independent experiments. Median values are shown

in box-and-whisker plots with whiskers designating minimum and maximum values. One-way ordinary ANOVA with Sidak's multiple comparison test was used to determine statistical significance. Only statistically significant comparisons are denoted. *, $P<0.05$

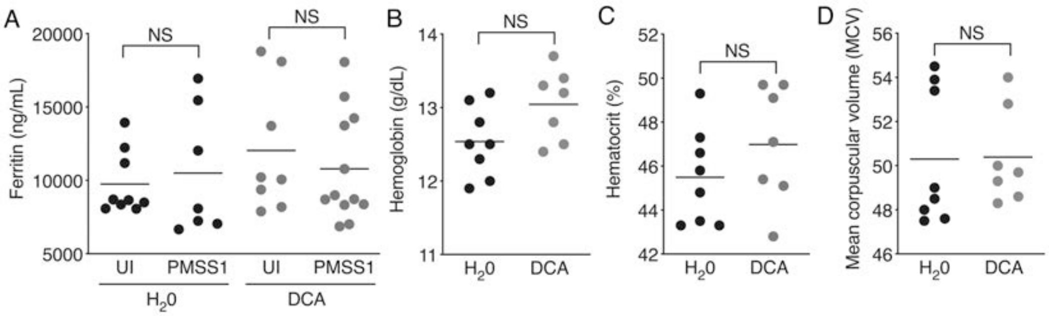

Supplemental Figure 12. DCA does not alter parameters of iron deficiency in INS-GAS mice. Male INS-GAS mice were maintained on an iron-replete standard diet and orogastrically challenged with Brucella broth (UI) or *H. pylori* strain PMSS1. Two weeks following infection, mice received water alone (H$_2$0) or water supplemented with 100 μM deoxycholic acid (DCA) throughout the course of the experiment. Mice were euthanized six weeks post-challenge. (**A**) Sera were was collected for ferritin ELISA and (**B-D**) whole blood was harvested for CBC analysis. Serum ferritin (*N*=36, **A**) hemoglobin (*N*=15, **B**), hematocrit (*N*=15, **C**), and mean corpuscular volume (*N*=15, **D**) were assessed as parameters of iron deficiency from a subset of mice from three independent experiments. Each point represents data from an individual animal and mean values are shown in scatter dot plots. One-way ordinary ANOVA with Sidak's multiple comparisons (**A**) and unpaired parametric t-tests (**B-D**) were used to determine statistical significance and none of the comparisons were statistically significant. NS, not statistically significant.

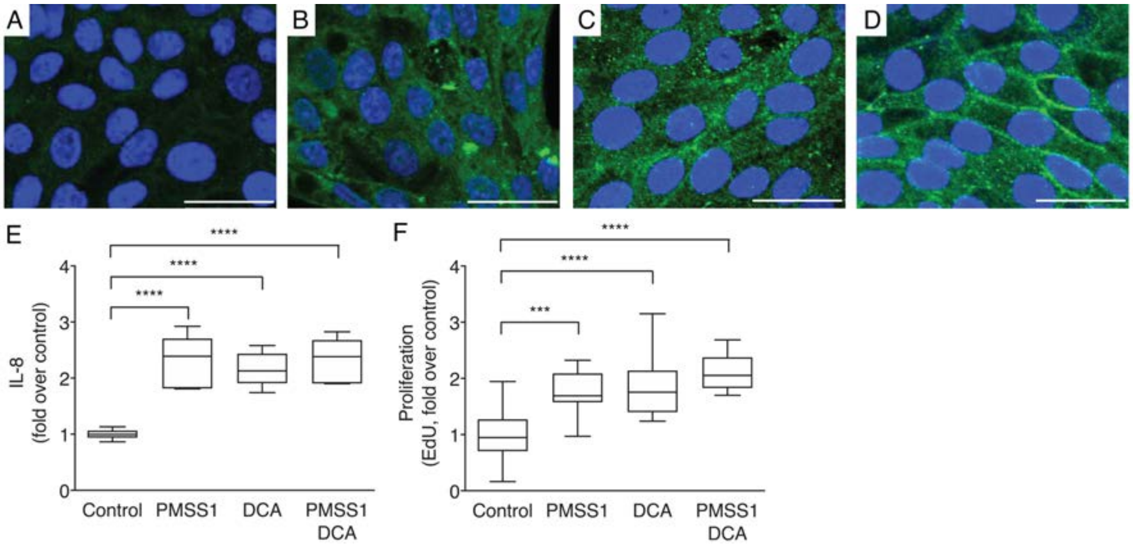

Supplemental Figure 13. ***H. pylori*** **infection and deoxycholic acid treatment augments pro-inflammatory and proliferative signaling pathways in human gastric organoids.** Primary human 2D gastric monolayers were co-cultured with or without *H. pylori* strain PMSS1 at a multiplicity of infection (MOI) of 10:1. Following bacterial adherence, monolayers were treated with or without 100 μM DCA and *H. pylori*-monolayer co-cultures were harvested 1 hour and 24 hours post-challenge. Gastric organoid co-cultures were assessed for p-EGFR by immunofluorescence (**A**, control; **B**, PMSS1; **C**, DCA; **D**, PMSS1 and DCA), IL-8 induction by ELISA (**E**), and proliferation as assessed via EdU immunofluorescence (**F**). Representative images are shown (400X). Scale bars represent 100 microns. Median values are shown in box-and-whisker plots with whiskers designating minimum and maximum values from three independent experiments. One-way ordinary ANOVA with Sidak's multiple comparison test was used to determine statistical significance. Only statistically significant comparisons are denoted. ****, $P<0.0001$; ***, $P<0.001$

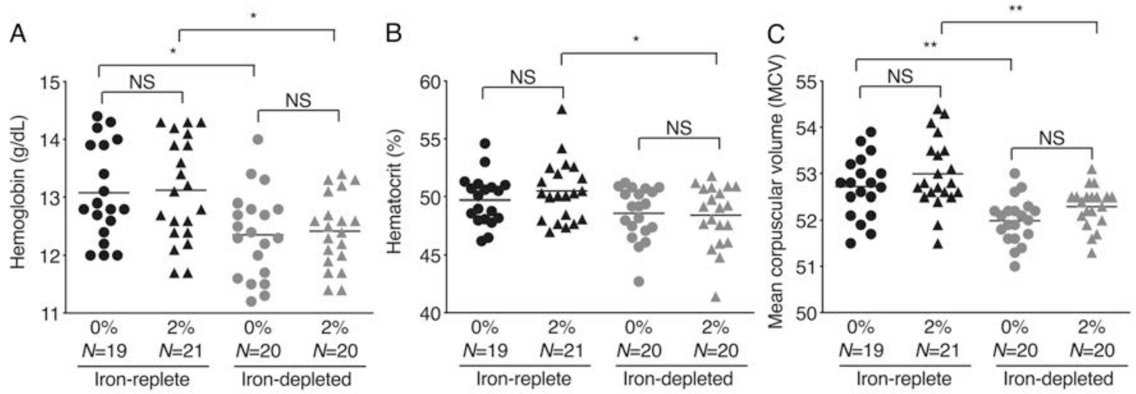

Supplemental Figure 14. Cholestyramine treatment does not affect parameters of iron deficiency. Male INS-GAS mice were maintained on iron-replete or iron-depleted diets supplemented with or without 2% cholestyramine (w/w) and then challenged with Brucella broth (UI) or *H. pylori* strain PMSS1. Mice were euthanized eight weeks post-challenge. Whole blood was collected for CBC analysis from a subset of *H. pylori*-infected mice maintained on iron-replete (*N*=40) or iron-depleted (*N*=40) diets with or without cholestyramine from three independent experiments. (**A**) Hemoglobin, (**B**) hematocrit, and (**C**) mean corpuscular volume were assessed as parameters of iron deficiency from three independent experiments. One-way ordinary ANOVA with Sidak's multiple comparisons test was used to determine statistical significance. Only statistically significant comparisons are denoted. **, $P<0.01$; *, $P<0.05$; NS, not statistically significant.

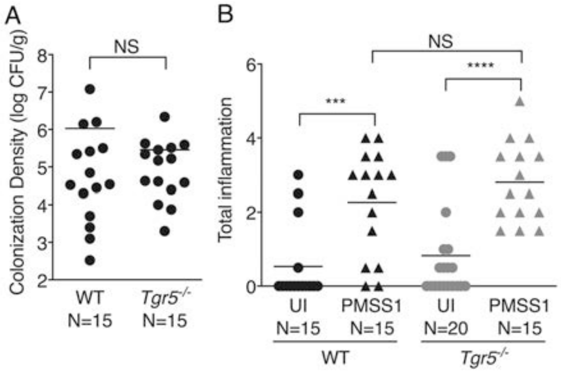

Supplemental Figure 15. Loss of *Tgr5* does not exert a significant effect on *H. pylori*-induced inflammation in C57BL/6 mice. Wild-type (WT) C57BL/6J (*N*=30) and *Tgr5*$^{-/-}$ homozygous (*N*=35) mice were maintained on iron-replete standard diet and were challenged with Brucella broth (UI) or *H. pylori* strain PMSS1. Mice were euthanized eight weeks post-challenge. (**A**) Gastric tissue was homogenized and plated for quantitative culture. Colonization density is expressed as log colony-forming units/gram of tissue. (**B**) Gastric tissue was fixed, paraffin-embedded, and stained with hematoxylin and eosin. Levels of total gastric inflammation (0-12) were assessed. Each point represents data from an individual animal from three independent experiments. Mean values are shown in scatter dot plots. Unpaired parametric t test (**A**) and one-way ordinary ANOVA with Sidak's multiple comparison test (**B**) were used to determine statistical significance. Only statistically significant comparisons are denoted. ****, $P<0.0001$; ***, $P<0.001$; NS, not statistically significant.
